# The Sexual Dimorphism in Rectum and Protein Digestion Pathway Influence Sex Pheromone Synthesis in Male *Bactrocera Dorsalis*

**DOI:** 10.1002/advs.202407353

**Published:** 2024-10-08

**Authors:** Jingxiang Chen, Yanling Jiang, Zijie Gao, Jiawang Dai, Chunsheng Jia, Yongyue Lu, Daifeng Cheng

**Affiliations:** ^1^ Department of Entomology South China Agricultural University Guangzhou 510640 China; ^2^ Guangdong Provincial Key Laboratory of Utilization and Conservation of Food and Medicinal Resources in Northern Region Shaoguan University Shaoguan 512005 China

**Keywords:** insect‐microbe interaction, rectum, sex pheromone, sexual dimorphism

## Abstract

Sexual dimorphism is a crucial aspect of mating and reproduction in many animals, yet the molecular mechanisms remain unclear. In *Bactrocera dorsalis*, sex pheromones trimethylpyrazine (TMP) and tetramethylpyrazine (TTMP) are specifically synthesized by *Bacillus* strains in the male rectum. In the female rectum, *Bacillus* strains are found, but TMP and TTMP are not, indicating sexually dimorphic differences in sex pheromone synthesis. Our anatomical observations and precursor measurements revealed significant differences in rectal structure and ammonium levels between sexes.  In vitro and in vivo experiments reveal that ammonium is vital for sex pheromone synthesis in rectal *Bacillus* strains. Comparative transcriptome analysis identified ammonium‐producing genes (carboxypeptidase B and peptide transporter) in the protein digestion pathway that show much higher expression in the male rectum than in the female rectum. Knocking down the expression of either carboxypeptidase B (or inhibiting enzyme activity) or peptide transporter decreases rectal ammonium levels significantly, resulting in the failure of sex pheromone synthesis in the male rectum. This study provides insights into the presence of sexual dimorphism in internal organs and their functionalities in male‐specific sex pheromone synthesis and has significant implications for understanding the molecular mechanisms underlying sex pheromone synthesis by symbionts in insects.

## Graphical abstract

A work model shows how protein digestion and absorption pathway influences male‐specific sex pheromone production by symbiotic *Bacillus sp*. in the rectum. The male‐specific rectal structure is different from that of female and the high expression of CPB and PEPT in the protein digestion and absorption pathway contribute to high levels of ammonium, leading to sex pheromone production by *Bacillus*.

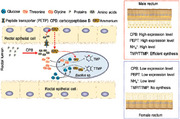

## 1 Introduction

Sexual dimorphism is a widespread phenomenon in animals due to the complex interplay of natural and sexual selection processes.^[^ ^1^ ^]^ In these species, males and females display varying morphological, physiological, and behavioral characteristics.^[^ ^2^, ^3^, ^4^ ^]^ Many avian species are known to exhibit sexual dimorphism: to attract mates, males typically display plumage that is more vibrant and vocalizations that are more melodious than those of females, and females may prioritize reproductive activities, such as nesting and incubating eggs.^[^ ^5^ ^]^ Similarly, in insects, to increase their likelihood of mating with females, males often exhibit brighter colors^[^ ^6^ ^]^ larger drum chambers,^[^ ^7^, ^8^ ^]^ and longer antennae^[^ ^9^, ^10^ ^]^ than females, and females usually actively seek suitable hosts for oviposition and reproduction.^[^ ^11^ ^]^ Additionally, certain male insects have specialized glands or cells dedicated to the production and dispersal of pheromones to attract females,^[^ ^12^, ^13^ ^]^ although sex pheromone compounds in insects are typically released by females to attract males for mating.^[^ ^14^ ^]^ Sexual dimorphism is influenced by many factors including genetics^[^ ^15^ ^]^ environmental conditions^[^ ^16^ ^]^ and endocrine regulation.^[^ ^17^ ^]^ The presence and evolution of sexual dimorphism result in distinct life histories between the sexes, enabling individuals to fulfill diverse roles in reproduction and survival.^[^ ^18^ ^]^ Notably, despite significant progress sexual dimorphism research, exploration of the molecular mechanisms responsible for these observed differences has been limited.

Insects exhibit sexual dimorphism in sex pheromone synthesis. For example, the generation of sex pheromones in insects is a complex process that differs between males and females. It involves multiple factors, such as production site and gene expression regulation. Females typically have specialized glands in the rear of their abdomen for pheromone production^[^ ^19^ ^]^ and males typically have secretion sites at various locations, such as the abdominal tip^[^ ^20^ ^]^ wings^[^ ^21^ ^]^ or prothorax.^[^ ^12^, ^22^ ^]^ These structural differences can affect pheromone function. Unique transcription factors and regulatory elements may show different activity levels in males and females, influencing the expression of genes related to pheromone production.^[^ ^23^ ^]^ Catalytic activities of metabolic enzymes may also exhibit variations.^[^ ^24^ ^]^ For instance, to generate sex pheromone precursors^[^ ^25^ ^]^ certain female insects depend on particular fatty acid oxidation pathways, and to produce alternative compounds, certain males utilize different routes than those found in females. In the past decades, studies have showed that the intestinal microbes are involved in the pheromone based communication process of animals. Before the 21st century, it was discovered that the gut microbes of beetles and locusts can produce pheromones that have important effects on host behavior.^[^ ^26^, ^27^ ^]^ Since then, researchers have found that microbes can affect kin recognition^[^ ^28^ ^]^ aggregation,^[^ ^29^, ^30^, ^31^ ^]^ development and olfactory behavior of insect hosts by producing the pheromone or influencing the pheromone producing.^[^ ^32^, ^33^ ^]^ Recently, more studies have found that symbiotic bacteria in beetles^[^ ^34^ ^]^ flies^[^ ^35^ ^]^ aphids,^[^ ^36^, ^37^ ^]^ and locusts^[^ ^38^, ^39^ ^]^ play crucial roles in pheromone synthesis. The presence of symbiotic bacteria significantly affects insect mating and reproductive behaviors.^[^ ^40^ ^]^ Although male and female insects carry similar bacteria, only one sex produces the corresponding pheromone, a phenomenon that is poorly understood.

**Figure 1:**
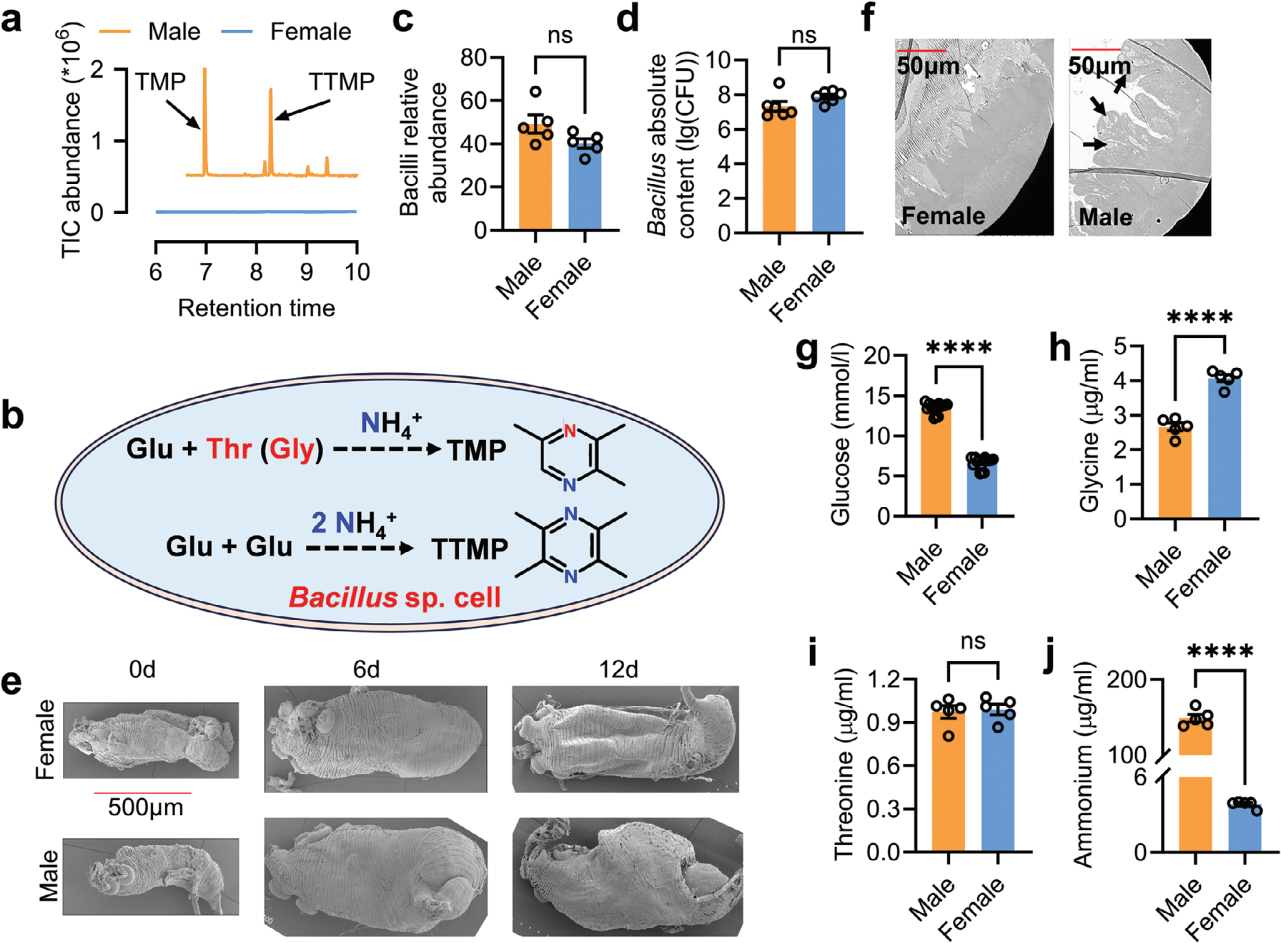
Sexual dimorphic sex pheromone, rectum morphology, and precursor levels between male and female. a) GC‐MS ion chromatograms of mature male and female rectum extracts. b) Schematic of sex pheromone synthesis in *Bacillus* cell. Glu: Glucose; Thr: Threonine; Gly: Glycine. c) Rectal Bacilli abundance comparison between mature males and females (n = 5, *P* = 0.0895, Independent sample *t*‐test). d) Rectal *Bacillus* absolute content comparison between mature males and females (n = 6, *P* = 0.1092, Independent sample *t*‐test). e) External morphological difference in male and female rectum. f) Internal morphological difference in male and female rectum. Black arrows indicate oily substances. g) Difference in rectum glucose levels between mature males and females (n = 15, *P* < 0.0001, Independent sample *t*‐test). h) Difference in rectum glycine levels between mature males and females (n = 5, *P* < 0.0001, Independent sample *t*‐test). i) Difference in rectum threonine levels between mature males and females (n = 5, *P* = 0.7706, Independent sample *t*‐test). j) Difference in rectum ammonium levels between mature males and females (n = 5, *P* < 0.0001, Independent sample *t*‐test).

In *B. dorsalis*, sex pheromones‐trimethylpyrazine (TMP) and tetramethylpyrazine (TTMP) are synthesized by *Bacillus* strains in the rectums of males.^[^ ^35^ ^]^ In the female rectum, *Bacillus* strains were found, and TMP and TTMP were not (**Figure** **1**), indicating typical sexually dimorphic differences in sex pheromone synthesis. In this study, the differences in rectal morphology and transcriptomes between males and females were compared. The results showed that male and female rectums exhibited significant morphological dimorphisms. Male specifically expressed genes in the protein digestion pathway play critical roles in generating rectal ammonium, which is critical to sex pheromone synthesis.

## 2 Results

### 2.1 Rectal Morphology and Sex Pheromone Precursor Levels may Contribute to male Specific Sex Pheromone Synthesis

In a previous study, we found that the male rectal *Bacillus* could produce the male specific sex pheromone‐TMP and TTMP.^[^ ^35^ ^]^ However, it is not clear why sex pheromone is specific to male, as female's rectum also contains a large number of bacteria. To answer such a question, we assessed the volatiles in the rectum of mature females and males and found that TMP and TTMP were only found in mature males (Figure 1a; Figure S1a,b, Supporting Information). As *Bacillus* uses glucose and amino acids (threonine or glycine) as substrates to synthesize TMP and TTMP with ammonium (Figure 1b)^[^ ^41^ ^]^ we can infer that differences in rectal morphology, *Bacillus* abundance, and glucose, amino acid, and ammonium levels between males and females may be the reason. To verify this hypothesis, we compared the rectal bacterial composition of mature males and females, and their rectal bacterial communities significantly differed (Figure S1c; Dataset S1, Supporting Information). However, there was no difference in Bacilli relative abundance and *Bacillus* absolute abundance (Figure 1c,d; Figure S1d, Supporting Information), indicating that rectal *Bacillus* might not contribute to male‐specific TMP and TTMP. Given that previous studies have confirmed that the specific structure of male rectum is the site where pheromones are synthesized and stored in other tephritid species.^[^ ^42^, ^43^, ^44^, ^45^, ^46^ ^]^ we then dissected and observed the external and internal morphologies of the male and female rectum. The results showed that the rectum of mature males was significantly enlarged compared with that of mature females (Figure 1e; Figure S1e,f, Supporting Information). The rectum of sexually mature males showed similar structure as *Bactrocera papaya* ^[^ ^44^ ^]^ including four papillae and an enlarged rectal sac which are absent in mature females (Figure S2a,c,e, Supporting Information). The rectum's internal structure showed that mature males had one more layer of cavity structure for storing sex pheromones than mature females, as seen in male of *B. papaya* ^[^ ^44^ ^]^ (Figure 1f; Figure S2b,d,f, Supporting Information). Therefore, the presence of rectal sac structures may be vital for sex pheromone synthesis. Next, rectal glucose, amino acid (Table S1, Supporting Information), and ammonium levels were compared between mature males and females, and significantly differences were observed (Figure 1g–j). Particularly, the rectal ammonium levels in mature males were 37 times higher than those in females (Figure 1j). Furthermore, ammonium levels in male rectum exhibited a more pronounced increase as male adults develop toward sexual maturity (Figure S1g, Supporting Information), in comparison to the fluctuations observed in the levels of *Bacillus* abundance and other precursors (glucose, glycine and threonine).^[^ ^35^, ^47^ ^]^ Together, these results suggest that differences in rectal morphology and substances may be the basis for male‐specific TMP and TTMP synthesis.

### 2.2 Influence of Different Precursor Levels on Sex Pheromone Production

**Figure 2:**
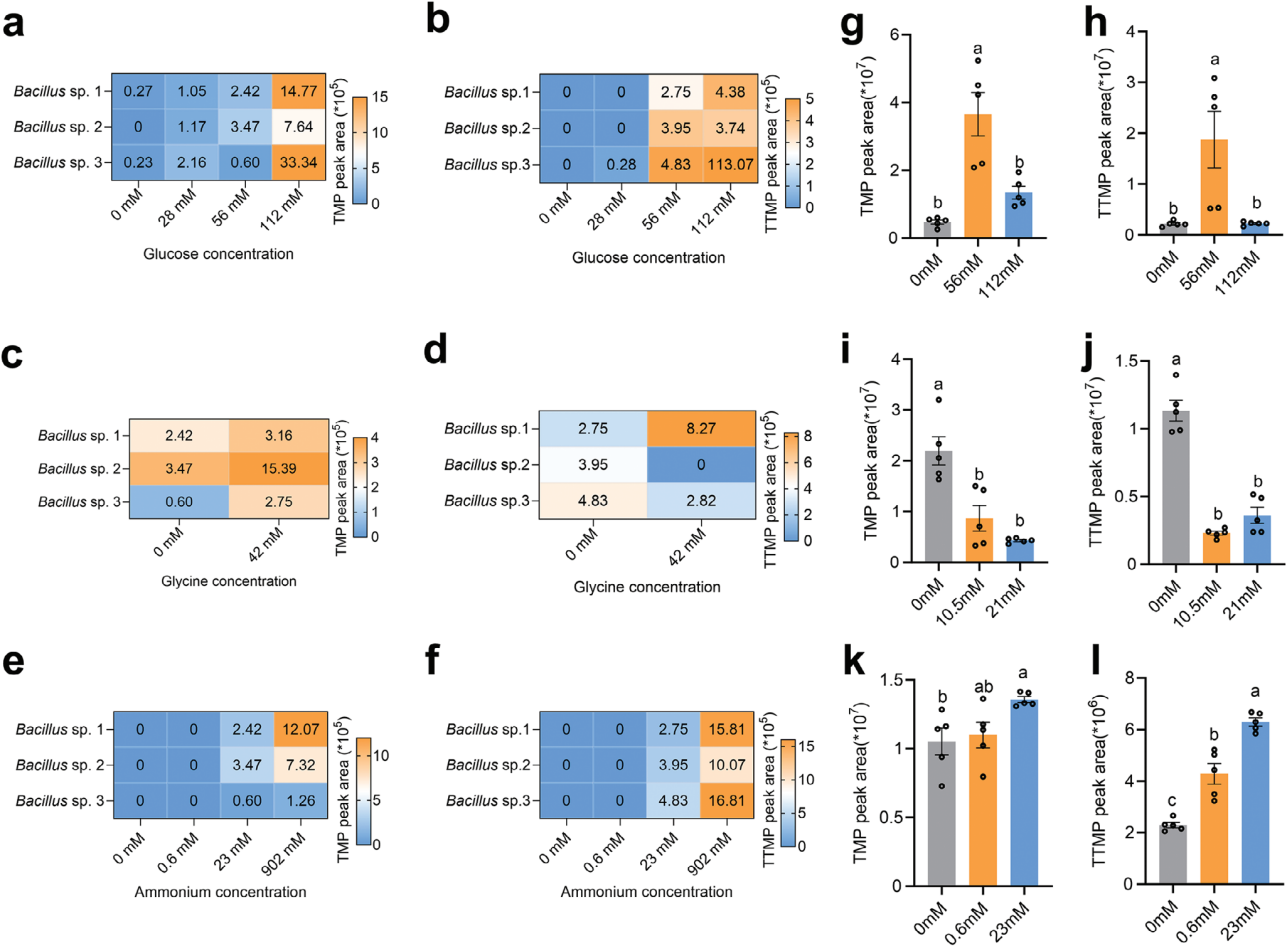
Influence of glucose, glycine, and ammonium levels on sex pheromone production by *Bacillus* strains isolated from mature male rectum. a) TMP produced by *Bacillus* in culture medium with different glucose levels. b) TTMP produced by *Bacillus* in culture medium with different glucose levels. c) TMP produced by *Bacillus* in culture medium with different glycine levels. d) TTMP produced by *Bacillus* in culture medium with different glycine levels. e) TMP produced by *Bacillus* in culture medium with different ammonium levels. f) TTMP produced by *Bacillus* in culture medium with different ammonium levels. g) TMP levels of synthesized by males fed with different glucose concentrations (n = 5, *F*_(2,12)_ = 18.14, *P* = 0.0002, one‐way ANOVA). h) TTMP levels of synthesized by males fed with different glucose concentrations (n = 5, *F*_(2,12)_ = 8.769, *P* = 0.0045, one‐way ANOVA). i) TMP levels of synthesized by males fed with different glycine concentrations (n = 5, *F*_(2,12)_ = 17.84, *P* = 0.0003, one‐way ANOVA). j) TTMP levels of synthesized by males fed with different glycine concentrations (n = 5, *F*_(2,12)_ = 74.16, *P* <0.0001, one‐way ANOVA). k) TMP levels of synthesized by males fed with different ammonium concentrations (n = 5, *F*_(2,12)_ = 4.320, *P* = 0.0386, one‐way ANOVA). l) TTMP levels of synthesized by males fed with different ammonium concentrations (n = 5, *F*_(2,12)_ = 61.75, *P* < 0.0001, one‐way ANOVA).

As glucose, glycine, and ammonium levels in the male and female recta significantly differed, we further tested the influence of different glucose, glycine, and ammonium levels on TMP and TTMP production by *Bacillus* ^[^ ^35^ ^]^ isolated from the male rectum in vitro. The different glucose, glycine, and ammonium levels significantly influenced TMP and TTMP production (**Figure** **2**). However, *Bacillus* was able to synthesize sex pheromones without glycine in the culture medium (Figure 2c,d; Figures S3g–i and S4m–r, Supporting Information), indicating glycine is not a factor affecting sex pheromone synthesis. For glucose, *Bacillus* could not synthesize TTMP but did synthesize a low amount of TMP in a culture medium without glucose (Figure 2a,b; Figures S3d–f and S4g–l, Supporting Information). What was noteworthy was that *Bacillus* could not synthesize sex pheromones in the culture medium with no ammonium or with low concentrations of ammonium (Figure 2e,f; Figure S3a–c, Supporting Information, Figure 3a–f). Because much higher ammonium levels were detected in the male rectum than in female (Figure 1i), the in vitro fermentation results suggest that different rectal ammonium levels may be a vital factor influencing male‐specific sex pheromone production.

**Figure 3:**
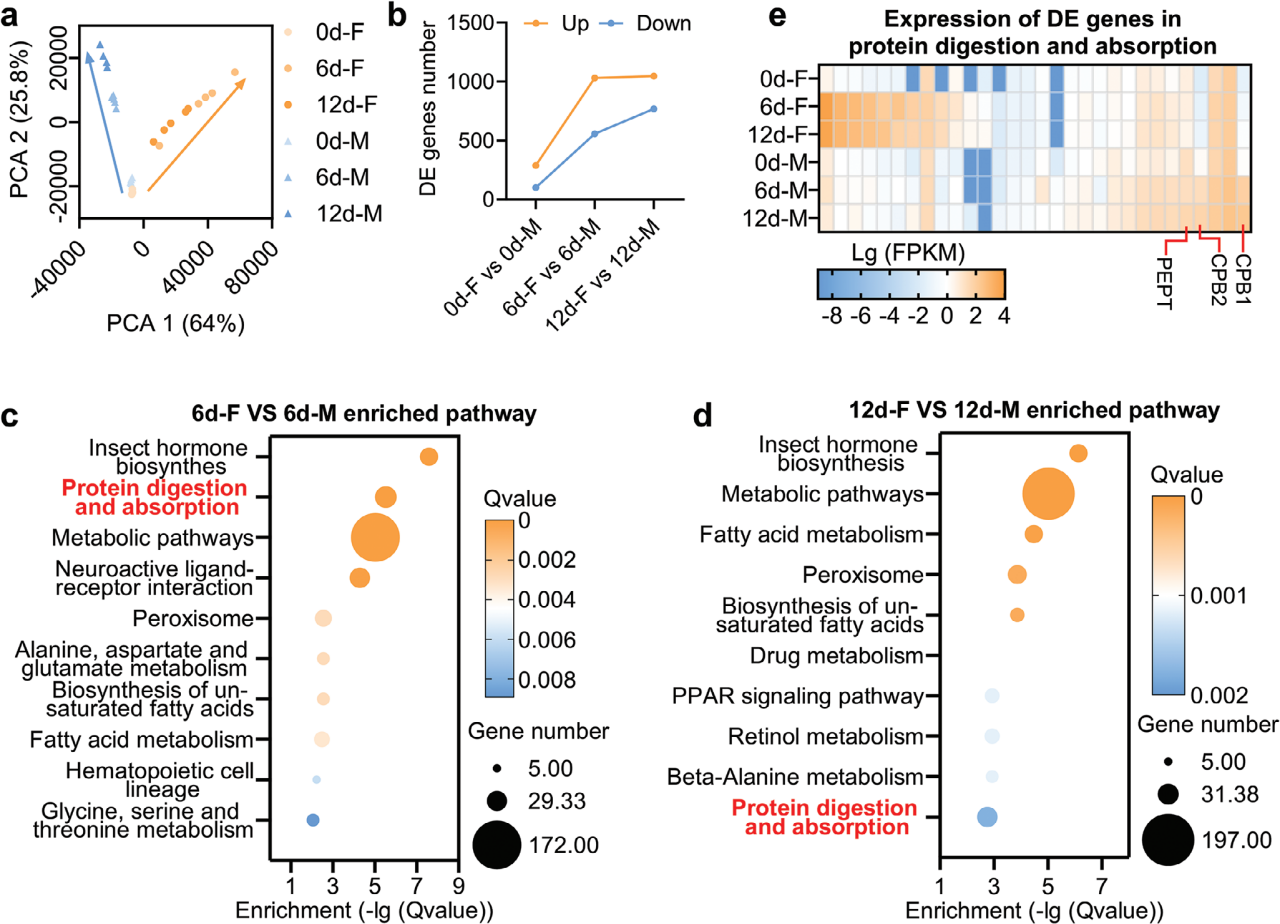
Rectal ammonium‐associated pathway and genes identification. a) Rectal gene expression patterns comparison between males and females at different ages by using principle component analysis. b) DEG number between male and female. c) KEGG pathways enriched with the rectal DEGs between 6‐day‐old males and females. d) KEGG pathways enriched with the rectal DEGs between 12‐day‐old males and females. e) Expression patterns of DEGs in the protein digestion and absorption pathway.

We further investigated the influence of different precursor levels on sex pheromone synthesis in vivo by feeding the males. The results showed that only the effect of ammonium on the synthesis of sex pheromone was concentration‐dependent which was consistent with the results in vitro (Figure 2k,l; Figure S3j, Supporting Information). However, high glucose concentration did not yield increased sex pheromone levels (Figure 2g–h; Figure S3k, Supporting Information). Furthermore, we found that the introduction of glycine led to a significant reduction in sex pheromone level (Figure 2i,j; Figure S3l, Supporting Information). In summary, our in vitro and in vivo investigations indicate that ammonium may be vital for sex pheromone synthesis.

### 2.3 Protein Digestion and Absorption Pathway may Contribute to Higher Ammonium Levels in the Rectum of Males than of Females

To further unravel the molecular basis of male‐specific sex pheromone production, a comparative transcriptome analysis between the male and female rectum was performed. Correlation analyses showed that the gene expression patterns of junior males and females were almost identical, whereas there were significant differences between senior males and females (**Figure** **3**a; Figure S5a; Dataset S2, Supporting Information). Differentially expressed gene (DEG) screening also showed that there were fewer DEGs between junior males and females than between their senior counterparts (Figure 3b; Figure S5b–d; Datasets S3–S5, Supporting Information). For screening the DEGs that may contribute to male‐specific sex pheromone synthesis, KEGG enrichment was performed using DEGs screened between males and females. The results showed that no pathway was significantly enriched with DEGs between 0‐day‐old males and females. However, various pathways were enriched with DEGs in the senior flies (Figure 3c,d; Datasets S6 and S7, Supporting Information). In particular, the protein digestion and absorption pathways were enriched in the 6‐day‐old and 12‐day‐old flies (Figure 3c,d). Because protein digestion and absorption pathways are involved in amino acid and ammonium metabolism,^[^ ^48^, ^49^ ^]^ we inferred that the genes in this pathway may be responsible for the higher rectal ammonium levels in males than in females. In the protein digestion and absorption pathway, the carboxypeptidase B (CPB1 and CPB2) and peptide transporter (PEPT) genes were highly expressed in the mature male rectum (Figure 3e; Dataset S7, Supporting Information). These results indicate that CPB and PEPT in the protein digestion and absorption pathways may be responsible for the higher rectal ammonium levels in males than in females, leading to sex pheromone synthesis.

### 2.4 CPBs Regulate Male Rectal Ammonium Level and Sex Pheromone Synthesis

**Figure 4:**
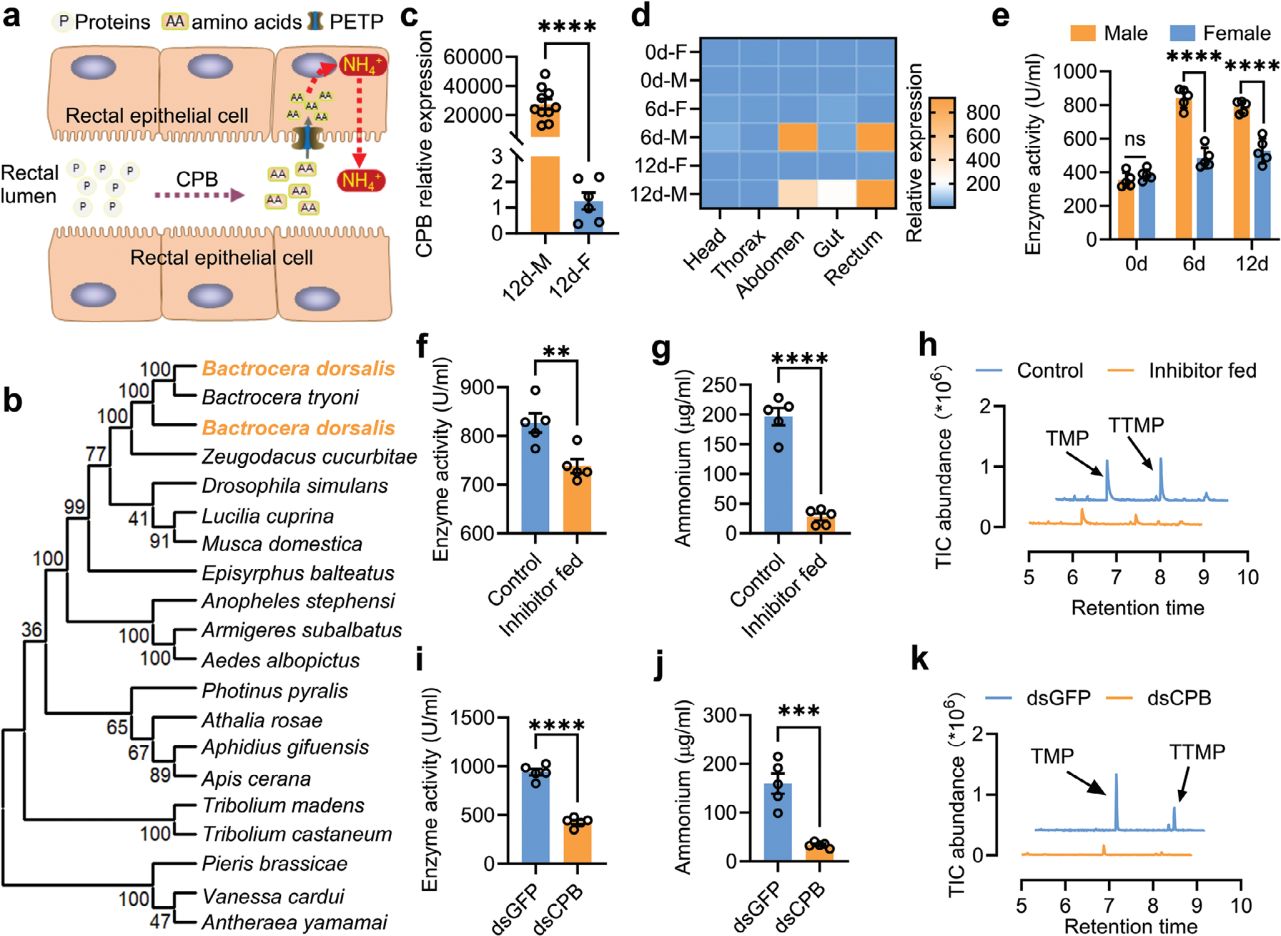
CPB influences sex pheromone synthesis by regulating rectal ammonium level. a) A hypothetical diagram showing that protein digestion and absorption pathway regulates rectal ammonium level. b) Maximum likelihood topology tree for diverse insect CPBs reveals conserved clades for CPB1 and CPB2 of *B. dorsalis*. c) CPB expression in rectum of mature males and females (n = 6 and 10, *P* < 0.0001, Independent sample *t*‐test). d) CPB expression in different tissues of mature males and females. e) CPB enzyme activities in males and females (0‐day old: n = 5, *P* = 0.23283, Independent sample *t*‐test; 6‐day old: n = 5, *P* < 0.0001, Independent sample *t*‐test; 12‐day old: n = 5, *P* = 0.0001, Independent sample *t* test). f) CPB enzyme activities in males with CPB being knocked down (n = 5, *P* = 0.0064, Independent sample *t*‐test). g) Influence of CPB inhibitor feeding on male rectal ammonium level (n = 5, *P* < 0.0001, Independent sample student's *t‐*test). h) GC‐MS ion chromatograms of rectum extracts showed influence of CPB inhibitor feeding on sex pheromone. i) Influence of CPB knocking down on rectal CPB enzyme activity of male (n = 5, *P* < 0.0001, Independent sample student's *t‐*test). j) Influence of CPB knocking down on male rectal ammonium level (n = 5, *P = *0.0003, Independent sample student's *t‐*test). k) GC‐MS ion chromatograms of rectum extracts showed influence of CPB knocking down on sex pheromone.

In the protein digestion and absorption pathway, CPB can catalyze the hydrolysis of proteins to produce free amino acids^[^ ^49^ ^]^ and then the free amino acids can be transported into the intestinal epithelial cell for further catabolism by PEPT^[^ ^48^ ^]^ (**Figure** **4**a). Therefore, we investigated the function of CPB in converting rectal free amino acids into ammonium. Maximum likelihood phylogenetic analysis using amino acid sequence alignments for 20 CPB culled from insects revealed that the CPBs were highly conserved (Figure 4b; Figure S6a, Supporting Information), which may indicate their similar roles in insects. Notably, the DNA sequences of CPB1 and CPB2 were almost 90% similar (Figure S7, Supporting Information). Therefore, we designed quantitative and RNA interference primers that act on both genes to study their functions (Table S2, Supporting Information). The qPCR results showed that the expression of CPB in the mature male rectum was 21861 times that in the females (Figure 4c). The investigation of tissue expression showed that CPB was mainly expressed in the rectum of mature males (Figures 4d; Figures S6b–S7d, Supporting Information). Moreover, CPB enzyme activity was significantly higher in the rectum of older males than in older females (Figure 4e). Potato carboxypeptidase inhibitor (PCI) was used as inhibitor^[^ ^50^ ^]^ of CPB and fed to the males. The results showed that PCI significantly decreased the CPB enzyme activity in males (Figure 4f). Consistently, rectal ammonium and sex pheromone levels decreased significantly (Figure 4g,h; Figures S8a and S9b, Supporting Information). The levels of sex pheromone precursors (glycine, threonine, and glucose) did not change in PCI‐fed males (Figure S8c–e, Supporting Information), although the levels of total amino acid and some other amino acids changed significantly (Figure S8f; Table S3, Supporting Information). After successfully decreasing the CPB expression in the rectum of mature males by RNAi (Figure S8g, Supporting Information), rectal CPB enzyme activity decreased significantly (Figure 4i), and the ammonium level decreased by more than four times (Figure 4j). Consistently, the rectal sex pheromone decreased significantly (Figure 4k; Figure S8h,i, Supporting Information). In addition, knocking down the expression of CPB did not affect the rectal threonine, glycine, or glucose levels (Figure S8j–l, Supporting Information), although the levels of total amino acids and some other rectal free amino acids changed significantly (Figure S8m; Table S4, Supporting Information). These results indicate that CPB in the protein digestion and absorption pathway can influence the rectal ammonium levels, which are vital for sex pheromone synthesis.

### 2.5 PEPT Regulates Male Rectal Ammonium Level and Sex Pheromone Synthesis

**Figure 5:**
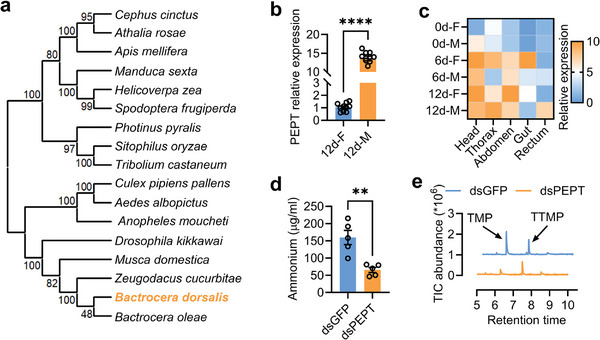
PEPT influences sex pheromone synthesis by regulating rectal ammonium level. a) A maximum likelihood topology tree for diverse insect PEPT reveals conserved clades for PEPT in Diptera. b) PEPT expression in rectum of mature males and females (n = 10, *P* < 0.0001, Independent sample *t*‐test). c) Tissue expression of PEPT in males and females. d) Rectum ammonium level comparison between PEPT knocked down mature male and control (n = 5, *P* = 0.0026, Independent sample *t*‐test). e) GC‐MS ion chromatograms show sex pheromone difference between PEPT knocked down mature males and control.

We also investigated the influence of PEPT on rectal ammonium. Maximum likelihood phylogenetic analysis using amino acid sequence alignments for 17 PEPT genes cultured from insects revealed that PEPT is conserved within insects (**Figure** **5**a; Figure S9a, Supporting Information). qPCR analysis showed that PEPT expression in the male rectum was 13 times higher than that in the female rectum (Figure 5b). Although tissue expression investigation results showed that PEPT was mainly expressed in the thorax of mature males, PEPT expression in the mature male rectum was much higher than that in females (Figure 5c; Figure S9b–d, Supporting Information). After successfully decreasing the expression of PEPT in the mature male rectum (Figure S10a, Supporting Information), the rectal ammonium level decreased almost three times (Figure 5d). Consistently, rectal sex pheromones decreased significantly (Figure 5e; Figure S10b,c, Supporting Information). Knockdown of PEPT expression did not affect rectal glucose levels (Figure S10f, Supporting Information), though threonine, glycine, and some other rectal free amino acid levels changed (Figure S10d,e; Table S5, Supporting Information). These results indicate that PEPT regulates sex pheromone synthesis by influencing rectal ammonium, threonine, and glycine levels.

## 3 Discussion

**Figure 6:**
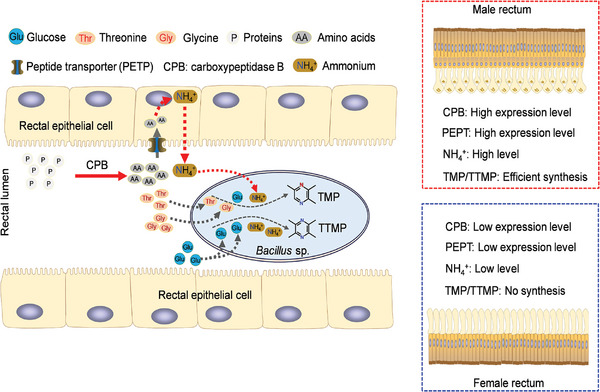
A work model shows how protein digestion and absorption pathway influences male‐specific sex pheromone production by symbiotic *Bacillus* sp. in rectum. The sexually mature male rectum has an extra cavity layer compared to mature females, potentially for storing sex pheromones. The high expression of the genes (*CPB* and *PEPT*) in the protein digestion and absorption pathway contribute to high levels of ammonium in the male rectum, leading to sex pheromone production by *Bacillus*.

In the realm of insect courtship and mating, there have been fascinating discoveries regarding the sex pheromone (pyrazines) synthesized by *Bacillus* in the male rectum.^[^ ^35^ ^]^ Specifically, the male rectal *Bacillus* needs to use glucose, amino acids and ammonium as precursors to synthesize sex pheromone.^[^ ^41^ ^]^ Although both sexes showed comparable levels of *Bacillus*, the *Bacillus* present in females exhibited a complete inability to produce pyrazines (Figure 1). Our study investigated the internal and external structures of the rectum of sexually mature males and females, revealing a more conducive structure for pheromone synthesis and storage in males than in females. Furthermore, we found that the male rectum specifically expressed CPB and PEPT, which play a role in protein digestion and absorption pathway, can affect synthesizing of male specific pyrazines by influencing the generation of rectal ammonium (**Figure** **6**). This study provides valuable insights into the molecular mechanisms underlying pyrazines synthesis by rectal *Bacillus*, shedding light on sexual dimorphism in internal organs and their functions.

Many insects utilize their rectum for water reabsorption^[^ ^51^ ^]^ and some fly species have turned their rectum into a perfume factory.^[^ ^42^, ^43^, ^45^, ^46^ ^]^ For example, male sex pheromones are produced in their enlarged rectum^[^ ^35^ ^]^ as observed in male *B. dorsalis*. Our study validated that dimorphic variances in sex pheromone‐producing organs determine the specificity of sex pheromones between males and females, revealing notable variations in the rectum between male and female *B. dorsalis*. Some male fruit flies exhibit a muscular rectum capable of expanding to accommodate the increased fluid volume and facilitate the rapid expulsion of its contents.^[^ ^44^ ^]^ These unique physiological attributes of insects may help them adapt to various reproductive environments and strategies and aid in sex recognition through structural differences.

Ammonium plays a crucial role in various processes in living organisms.^[^ ^35^, ^52^ ^]^ The literature on *Bacillus* species has highlighted the importance of ammonium as a critical substrate for TMP/TTMP synthesis.^[^ ^41^, ^53^ ^]^ Imbalances in ammonium levels impede *Bacillus* growth and TMP/TTMP synthesis.^[^ ^54^ ^]^ In the male rectum of *B. dorsalis*, there was no significant difference in *Bacillus* abundance; however, the ammonium content was significantly higher in males than in females, implying that ammonium in the male rectum may be used to synthesize sex pheromones. High levels of ammonium may not only serve as a nitrogen source for sex pheromone synthesis but also create an alkaline environment in the rectum, potentially enhancing catalytic reactions. For example, the optimum pH for TTMP formation is 7.5 in *Bacillus subtili*.^[^ ^55^ ^]^ However, this hypothesis requires further testing.

After emergence, *B. dorsalis* undergoes a crucial feeding phase to reach sexual maturity. This period is essential for growth and reproduction and requires the digestion and absorption of key nutrients.^[^ ^47^, ^56^ ^]^ Our study revealed a noteworthy surge in glucose levels within the rectum of sexually mature males compared with those of females, suggesting that males may engage in more energy‐intensive processes than females do. Glucose is a crucial precursor in the synthesis of TMP/TTMP.^[^ ^35^ ^]^ The rectum stores and eliminates undigested matter and waste products.^[^ ^57^ ^]^ The dissection of sexually mature males and females revealed a stark contrast in the rectum. The rectum of females exhibited a build‐up of unmetabolized yellow yeast powder, and the rectum of males remained transparent with no visible residue (Figure S11, Supporting Information). This disparity indicates that males have a stronger ability to metabolize yellow yeast powder than females do. In addition, the rectum of males at the sexually mature stage was significantly enriched in protein digestion and absorption pathway. This metabolic process generates ample amino acids or ammonium,^[^ ^48^, ^49^ ^]^ which can be further utilized by *Bacillus* spp. to synthesize sex pheromones. CPB is involved in degrading and releasing free amino acids one by one from the C‐terminus of the peptide chain. The lack of CPB in females results in the inability of the enzyme to hydrolyze proteins into free amino acids^[^ ^49^ ^]^ similar to the decrease of total amino acids content seen in male rectum after CPB disruption or inhibition. Consequently, free amino acids cannot be absorbed by intestinal epithelial cells for subsequent catabolism by PEPT.^[^ ^48^ ^]^ Given that ammonium in organisms primarily originated from amino acid, a reduction in the overall amino acid content may result in decreased in ammonium content. Thus, the female rectum does not have the ability to produce precursors of TMP/TTMP, such as ammonium. This finding suggests that variations in host gene expression influence the metabolic activity of symbiotic bacteria. Subsequent studies should focus on identifying host genes that govern microbial metabolism. Manipulating these genes might modify microbial metabolism, realizing the goal of using bacteria to control pests.

Nevertheless, this study also has certain limitations concerning its methodology. Landscapes of some other amino acids also differed significantly in males compared with females, CPB inhibitor feeding males, CPB knocked down males or PEPT knocked down males (Tables S1 and S3–S5, Supporting Information). Because our research focused on the influence of ammonium on pheromone synthesis, we did not investigate the potential functions of these amino acids. The potential biological functions can be further investigated in the future. Furthermore, the precise mechanism of ammonium production in intestinal cells and the associated transporter process will be validated in forthcoming research endeavors. Although RNAi and inhibitors were effectively employed to reduce mRNA and enzyme activity of the target genes, the lack of an in vivo overexpression system (e.g., the GAL4 /UAS system) in this non‐model species impeded our capacity to investigate effect of gene overexpression on sex pheromone production. Furthermore, the process by which the sex pheromone is conveyed and retained in the rectum of sexually mature male is not yet fully understood. The investigation of the synthesis, transport, storage, and release of pheromones in living organisms through the utilization of isotope‐labeling could offer significant insights into these biological processes.

## 4 Experimental Section

### Insects

The *B. dorsalis* strain obtained from a carambola (*Averrhoa carambola*) orchard in Guangzhou, Guangdong Province, was reared in controlled laboratory conditions (27 ± 1 °C, 12:12 h light: dark cycle, 70–80% RH). Larvae were nourished with a maize‐based artificial diet comprising 150 g corn flour, 150 g banana, 0.6 g sodium benzoate, 30 g yeast, 30 g sucrose, 30 g paper towel, 1.2 mL hydrochloric acid, and 300 mL water. Adults were provided with a solid diet (i.e., 50 g yeast and 50 g sugar) and 50 mL sterile water within a 35 cm × 35 cm × 35 cm wooden cage. In *B. dorsalis*, male and female flies reach sexual maturity and begin mating 12 d after emergence. To ensure the inclusion of unmated individuals in the study, female and male flies were selected for separate rearing 6 d after emergence.

### Rectum Morphology Observation

For the rectal size comparison, dissections were performed on the rectum of sexually mature males and females (12 days old). The dissected rectums were placed under a microscope for observation and measurement of their length and width.

For scanning electron microscopy (SEM), the dissected rectums were initially fixed in 2.5% glutaraldehyde for 4 h, followed by rinsing with 0.1 M PBS for 40 min. Subsequently, the specimens were fixed with 1% osmium acid for 1 h and rinsed with 0.1 M PBS buffer. The samples were dehydrated through a series of ethanol gradients (30, 50%,70%, 80, 90, and 100%) for 10 min per treatment to ensure complete removal of water. Critical point desiccation was employed to dry the samples, followed by gold spraying and the subsequent examination and photographic documentation by using a scanning electron microscope.

For transmission electron microscopy (TEM), the rectums were fixed in a solution containing 2.5% glutaraldehyde and 2% paraformaldehyde for 4 h, followed by a 1 h rinse with 0.1 M PBS. Samples were fixed in 1% osmium acid for 2 h and rinsed with double‐distilled water for 1 h. Staining was performed overnight using a bisulfite acetate block, and the subsequent dehydration was performed using graded ethanol (30%, 50%,70%, 85%, 95%, and 100%) for 15 min per treatment to ensure the complete removal of water from the samples. The specimens were embedded in a resin embedding agent and processed for post‐sectioning staining with an ultramicrotome; next, a transmission electron microscope was used for observations and to capture images.

### Sex Pheromone Identification in Rectum

After the dissection of 60 rectums, the specimens were placed in a sterile, enzyme‐free brown chromatography vial with 500 µl chromatographically pure hexane. Subsequently, they were incubated in a low‐temperature shaking incubator for 24 h at 16 °C and 200 rpm. The resulting supernatant was transferred to a separate sterile enzyme‐free brown chromatography vial. Each group of samples was replicated three times. The samples were analyzed using an Agilent gas chromatography‐mass spectrometer (GC‐MS), and the experimental parameters were as previously described.^[^ ^47^ ^]^ Finally, the peak areas of both TMP and TTMP were determined.

### Determination of Rectal Bacterial Diversity

To investigate the alterations in bacterial populations within the rectum of sexually mature females and males, the composition of the gut microbiota was analyzed. Rectums from eight individuals were collected and processed by extracting rectal bacterial DNA using a Bacterial Genomic DNA Extraction Kit (Tiangen, Beijing, China) according to the manufacturer's protocol. Subsequently, PCR amplification was conducted using universal primers 1492R and 27F. The resulting PCR products that fulfilled the specified criteria were outsourced for sequencing to Guangzhou Kidio Biotechnology Co., Ltd., with sequencing targeting the V3–V4 region. Each experimental group was analyzed using five biological replicates. The 16S rRNA sequencing data were subjected to preprocessing and clustering of valid sequences by using the UPARSE software sequence clustering algorithm. Sequences exhibiting over 97% similarity were grouped into operational taxonomic units (OTUs), and representative sequences were compared with a reference database for taxonomic classification. Using R language, a principal component analysis plot was generated.

### Rectum *Bacillus* Absolute Content Measurement

Considering that 16r RNA amplicon sequencing can't reflect the absolute amount of *Bacillus* in rectum, rectal *Bacillus* absolute contents of mature male and female were measured by qPCR. Briefly, 5 rectums were collected as one sample. Then, the bacterial DNA in the rectum was extracted using the Bacterial Genomic DNA Extraction Kit (Tiangen, Beijing, China, http://www.tiangen.com/asset/imsupload/up0250002001571219042.pdf) according to the manufacturer's protocol. qPCR was used to estimate the differences in the absolute abundance of *Bacillus*. Primers targeting the 16S rRNA gene of *Bacillus* were designed to amplify the gene from rectal *Bacillus* (Table S2, Supporting Information). Before amplification, a standard curve to quantify Bacillus abundance was generated. Briefly, the genomic DNA of *Bacillus* ^[^ ^35^ ^]^ isolated from mature males was extracted for amplification with the primer (Table S2, Supporting Information). The amplified fragment was then cloned and inserted into the pMD 18–T vector, which was then transferred into *E. coli* DH5α for propagation. The propagated vector was then extracted with a plasmid extraction kit and subjected to 10‐fold serial dilution to obtain 5 different plasmid concentrations (measured by a Nanodrop spectrophotometer). Then, a standard curve for quantifying Bacillus abundance was generated by amplifying the 16S rRNA of the plasmid. By referring to the standard curve, the absolute abundance of *Bacillus* in rectum was determined.

### Rectal Glucose Level Measurement

The method for measuring rectal glucose levels was described in Gui et al. (2023).^[^ ^47^ ^]^ In brief, rectums obtained from 15 sexually mature individuals of *B. dorsalis* were meticulously dissected under a microscope and transferred to a centrifuge tube containing 15 µl enzyme‐free sterile water. Subsequently, the samples were centrifuged at 12,000 rpm for 10 min after the addition of glass beads and thorough grinding. Finally, the glucose levels in the supernatant were measured using a glucometer (ONETOUCH, Verio Flex).

### Rectal Amino Acid and Ammonium Levels Measurement

For determining rectal threonine, glycine, and ammonium levels, samples were prepared for free amino acid analysis by using the protocol of Shahzad et al. (2019).^[^ ^58^ ^]^ Briefly, 30 rectums obtained from sexually mature males and females were microscopically dissected and subsequently transferred into a centrifuge tube containing 500 mL of a 5% sulfosalicylic acid solution. Glass beads were introduced into the tube, the contents were thoroughly ground, and the mixture stood for 1 h. Subsequently, the sample was centrifuged at 12,000 rpm for 10 min, and the resulting supernatant was filtered through a 0.22 µm filter tip into a liquid‐phase injection vial. The filtered sample was quantified using an amino acid analyzer (Hitachi L‐8900, Japan) according to the standard method. In amino acid analyzers, the peak area is generally proportional to the content of each sample component. This study compared total amino acid content based on the total peak area.

### Rectal *Bacillus* Cultivation Experiments

To investigate the influence of different glucose, glycine, and ammonium levels on the production of TMP/TTMP by rectal *Bacillus*, a series of in vitro fermentation experiments were conducted. Three distinct TMP/TTMP‐producing *Bacillus* strains previously isolated from male rectum^[^ ^35^ ^]^ were utilized for this purpose. *Bacillus pumilus*, *Bacillus altitudinis*, and *Bacillus safensis* are designated as *Bacillus* sp.1, *Bacillus* sp.2, and *Bacillus* sp.3 in this study. Basic media containing glucose (56 mm), threonine (42 mm), and (NH_4_)_2_HPO_4_ (23 mm) were prepared. In the glucose experimental group, four concentrations were set to 0, 28, 56 (basic medium), and 112 mm. In the ammonium experimental group, four concentrations were used: 0, 0.6, 23 (basic medium), and 902 mm. In the glycine experimental group, the concentrations were set to 0 (basic medium) and 42 mm. These substances were reconstituted using sterile water, added to 250 mL liquid LB medium through a 0.22 µm bacterial filter, and then 50 µL of the bacterial solution was introduced. The mixture was incubated in a constant temperature shaker at 37 °C and 180 rpm min^−1^ for 3 d. The volatiles were then extracted by headspace extraction using solid‐phase microextraction (SPME). Specific methodologies, mass spectrometry, gas chromatography parameters, and standards for characterizing the volatiles were adopted from Gao et al. (2023).^[^ ^40^ ^]^

### Sex Pheromone Identification in Male Rectum after Feeding on Ammonium, Glucose, and Glycine

To investigate the influence of different glucose, glycine, and ammonium levels on the production of TMP/TTMP in vivo, a series of feeding experiments were conducted. In the glucose experimental group, three glucose concentrations were set to 0, 56, and 112 mm. In the ammonium experimental group, three ammonium concentrations were used: 0, 0.6, and 23 mM. In the glycine experimental group, the glycine concentrations were set to 0, 10.5, and 21 mm. A post‐starvation feeding protocol was implemented in this study, wherein sexually mature males were subjected to a 12 h fasting period. Adults received a basic diet of yeast and sucrose, supplemented with aqueous solutions of various concentrations for 12 h. Rectums were dissected and analyzed for sex pheromone (TMP/TTMP) content using GC/MS, and the experimental parameters were as previously described.^[^ ^47^ ^]^ Finally, the peak areas of both TMP and TTMP were determined.

### Rectal Transcriptome Sequencing

To identify the pathways or genes that exhibited differential expression and potentially affected variations in the aforementioned substances at the genetic level, we conducted a comparative analysis of the transcriptome sequencing results of male and female rectal samples at 0, 6, and 12 d time intervals. Junior refers to individuals who are not sexually mature (both 0d and 6d), while senior refers to individuals who are sexually mature (12d). Five rectal specimens were dissected for RNA extraction from each experimental group, with five replicates per group. Subsequently, paired‐end RNA‐seq libraries were prepared using the Illumina library construction protocol and sequenced using the Illumina HiSeq2000 platform (Illumina, USA). For subsequent analysis, raw reads in the FASTQ format were generated and sorted based on barcodes. Before assembly, raw paired‐end reads from each cDNA library were preprocessed to remove adaptors, low‐quality sequences (Q < 20), and reads containing microbial contaminants. The clean reads were assembled de novo to produce contigs. An index of the reference genome of *B. dorsalis* was established, and paired‐end clean reads were aligned to the reference genome by using HISAT2. 2.4 was executed with parameters that included rna‐strandness RF and the default settings.^[^ ^59^ ^]^ To assess transcript expression abundances, we used StringTie software to calculate the normalized gene expression value of FPKM.^[^ ^60^ ^]^ Subsequently, DESeq2 software was used to analyze differential gene expression.^[^ ^61^ ^]^ Genes or transcripts fulfilling the criteria of a false discovery rate below 0.05 and an absolute fold change of > 2 were identified as DEGs or transcripts. Sample correlation analysis was performed using the statistical software R to compute the correlation coefficient between sample pairs to evaluate their degree of similarity. A correlation coefficient closer to 1 indicates a higher degree of similarity between samples. Principal component analysis was conducted using the R package gmodels to clarify the structure and interrelationships among the samples. Moreover, pathway enrichment analysis was conducted to identify significantly enriched metabolic or signal transduction pathways in differentially expressed genes compared with those in the entire genome.

### Phylogenetic Sequence Analysis

Phylogenetic analysis was conducted using the amino acid sequence alignments of 20 CPB and 17 PEPT sequences identified in insects. Amino acid sequence analysis was performed using MEGA11, and maximum likelihood (ML) tree reconstruction was performed using the Poisson model and uniform rates. The ML heuristic search was performed using the nearest neighbor‐change method, and the initial tree was selected by applying the neighbor‐joining method to a matrix of pairwise distances estimated using the JTT method. The accuracy of the tree was tested using bootstrapping with 100 replicates. The conservation of CPB and PEPT proteins was determined using the WebLogo tool (https://weblogo.berkeley.edu/logo.cgi).

### CPB Enzyme Activity Measurement

Twenty rectums were collected and placed in a 1.5 mL centrifuge tube with 30 µL 0.9% saline. The samples were homogenized and centrifuged at room temperature (3000 rpm for 10 min). The resulting supernatant was then transferred to a new centrifuge tube and preserved at −20 °C for subsequent analysis. Five biological replicates were collected during each sampling period.

An ELISA kit for Insect Carboxypeptidase B (AllendaBio, Shanghai, China) was used to measure the enzyme activity in the samples according to the manufacturer's instructions. The optical density (OD) of the samples was assessed by allowing them to equilibrate at room temperature for 20 min before commencing the assay. Standard wells were loaded with 50 uL of standards containing different concentration gradients (0, 50, 100, 200, 400, 800 U mL^−1^), and sample wells received 10 µL of the test sample and 40 µL sample diluent, with blank wells remaining unaltered. Subsequently, 100 µL HRP‐labeled antibody was added to each standard and sample well. The reaction plate was then sealed with film and incubated at 37 °C for 60 min, and excess liquid was removed and blotted dry on absorbent paper. The washing solution provided by the kit was added to each well, allowed to stand for 1 min, shaken off, and blotted dry on absorbent paper. This process was repeated five times. Subsequently, 50 µL of substrate A and B (provided by the kit) were added to each well, followed by a 15 min incubation at 37 °C in the absence of light. Finally, 50 µL termination solution (provided by the kit) was added to each well, and the OD value was measured at 450 nm. A regression curve was fitted for the standard, with concentration as the horizontal coordinate and the OD value as the vertical coordinate (R^2^ > 0.99). The enzymatic activity of the CPB was assessed by measuring the OD of the sample and correlating it with a regression curve.

### Tissue Expression of CPB and PEPT

Quantitative real‐time PCR (qRT‐PCR) was used to study the expression patterns of CPB and PEPT genes in various tissues. RNA was extracted from different specimens was performed using TRIzol reagent, followed by cDNA synthesis using the One‐Step gDNA Removal and cDNA Synthesis SuperMix Kit (TransGen Biotech). qRT‐PCR was performed using the PerfectStarTM Green qPCR SuperMix Kit (TransGen Biotech) to quantify gene expression levels. Gene‐specific primers for target genes (Table S2, Supporting Information) were designed using Primer BLAST on the NCBI platform. The reference genes used in this study were *α‐tubulin* and *RPL60*.^[^ ^62^ ^]^

### CPB Inhibitor Assay

PCIs have been effectively used in insects to suppress the activity of the CPB enzyme.^[^ ^50^ ^]^ A PCI inhibitor obtained from Shanghai Yingxin Laboratory Equipment Co., Ltd. was used to inhibit CPB enzyme activity in *B. dorsalis*. A post‐starvation feeding protocol was implemented in this study^[^ ^63^ ^]^ wherein sexually mature males were subjected to a 24 h fasting period. Subsequently, the inhibitor was administered at a concentration of 50 ug mL^−1^ in a volume of 1 mL for the duration of 6 h starting at 8:00 the next day, before resuming a normal diet. The effect of the inhibitor on the levels of rectal glucose, amino acids, ammonium, and sex pheromones was assessed.

### RNAi Assays

The dsRNA primers with the T7 promoter sequence were custom designed based on the Coding DNA Sequences (CDSs) of PEPT and CPB (Table S2, Supporting Information). To avoid off‐target effect in RNAi, the online tool (Dsomg.sysu.edu.cn) was used to perform off‐target prediction of RNAi target sites in *B. dorsalis* by setting the threshold of off‐target bases to 19 bp.^[^ ^64^ ^]^ Synthesis and purification of dsRNA were performed using a MEGAscript RNAi Kit (Thermo Fisher Scientific) per the manufacturer's instructions. The GFP gene (GenBank accession number: AHE38523) served as a negative control for the RNAi experiments. In males, 0.5 µl dsRNA at a concentration of 500 ng µl^−1^ was injected into the abdomen of 12‐day‐old individuals to target PEPT and CPB. Flies injected with dsGFP, double‐stranded RNA derived from the green fluorescent protein gene, were used as the control group. Subsequently, the knockdown efficiency of the targeted genes was assessed after 24 h by using quantitative real‐time polymerase chain reaction (RT‐qPCR) per established protocols for gene expression validation. The impact of PEPT and CPB silencing on the levels of glucose, amino acids, ammonium and sex pheromones was evaluated. Enzyme activity subsequent to CPB interference was assessed in accordance with the guidelines provided by the ELISA kit.

### Data Analysis

Statistical analysis methods used in the study were indicated in the figure legends. Differences were considered significant when *P* < 0.05. All data were analyzed using the GraphPad Prism version 10, GraphPad Software, La Jolla, CA, USA, https://www.graphpad.com/.

## Conflict of Interest

The authors declare no conflict of interest.

## Supporting information



Supporting Information

Supplemental Dataset 1

Supplemental Dataset 2

Supplemental Dataset 3

Supplemental Dataset 4

Supplemental Dataset 5

Supplemental Dataset 6

Supplemental Dataset 7

Supplemental Dataset 8

## Data Availability

Research data are not shared.
