# Supplementary material for: The Sexual Dimorphism in Rectum and Protein Digestion Pathway Influence Sex Pheromone Synthesis in Male Bactrocera Dorsalis
Source: Adv Sci (Weinh). 2024 Oct 8;11(44):2407353. doi: 10.1002/advs.202407353 (PMC11600207; doi:10.1002/advs.202407353)
Supplement: Supplementary file 1 — Supporting Information [file ADVS-11-2407353-s007.docx]

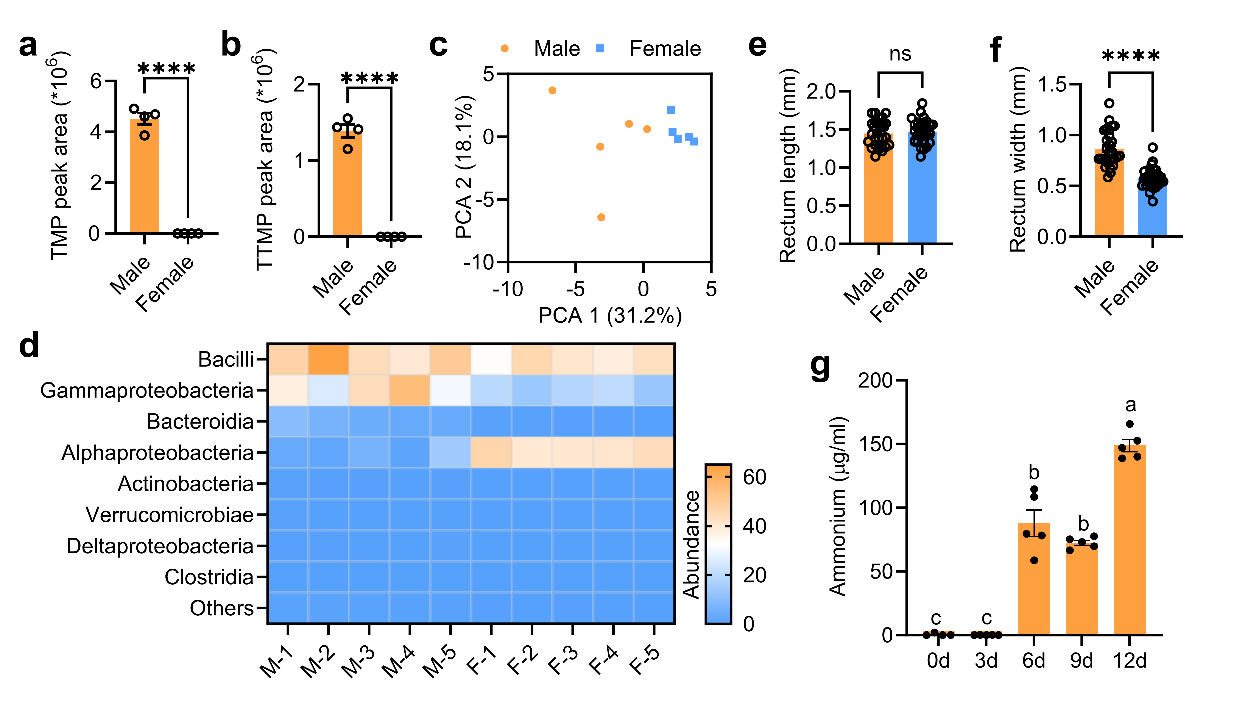


**Figure S1 Rectal sex pheromone level, bacteria diversity and morphology difference between mature male and female.** (a) Difference of TMP contents between mature male and female (n = 4, *P* < 0.0001, Independent sample *t* test). (b) Difference of TTMP contents between mature male and female (n = 4, *P* < 0.0001, Independent sample *t* test). (c) Rectal bacteria diversity comparison by principal component analysis (PCA) between mature male and female. (d) Rectal bacteria abundance in mature male and female. (e) Difference of rectum length between mature male and female (n = 29 and 31, *P* = 0.5924, Independent sample *t* test). (f) Difference of rectum width between mature male and female (n = 29 and 31, *P* < 0.0001, Independent sample *t* test). (g) Changes of ammonium content in males at different development stages (n = 5, *F* (4,19) = 132.5, *P* <0.0001, one-way ANOVA).

**
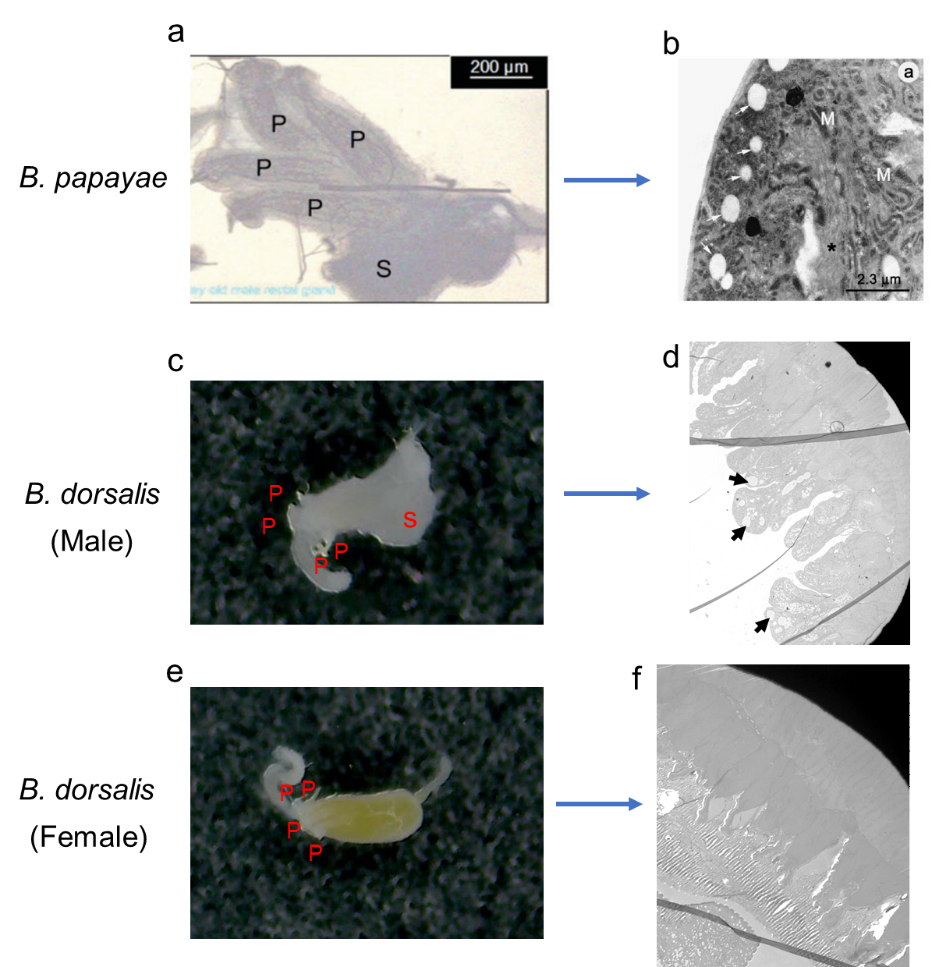
**

Figure S2 Comparison of the internal and external morpho-anatomy of the rectum in *Bactrocera papayae* and *Bactrocera dorsalis*. (a) Morphologic diagram of the rectum of male *B. papaya*. (b) Oily droplet material appears in the rectum of male *B. papayae*^1^. White arrows label oily droplets. (c) Morphologic diagram of the rectum of male *B. dorsalis*. (d) Oily droplet material appears in the rectum of male *B. dorsalis*. (e) Morphologic diagram of the rectum of female *B. dorsalis*. (f) No oily droplet material appears in the rectum of female *B. dorsalis*. Black arrows label oily droplets. S: rectal sac, P: rectal papilla.


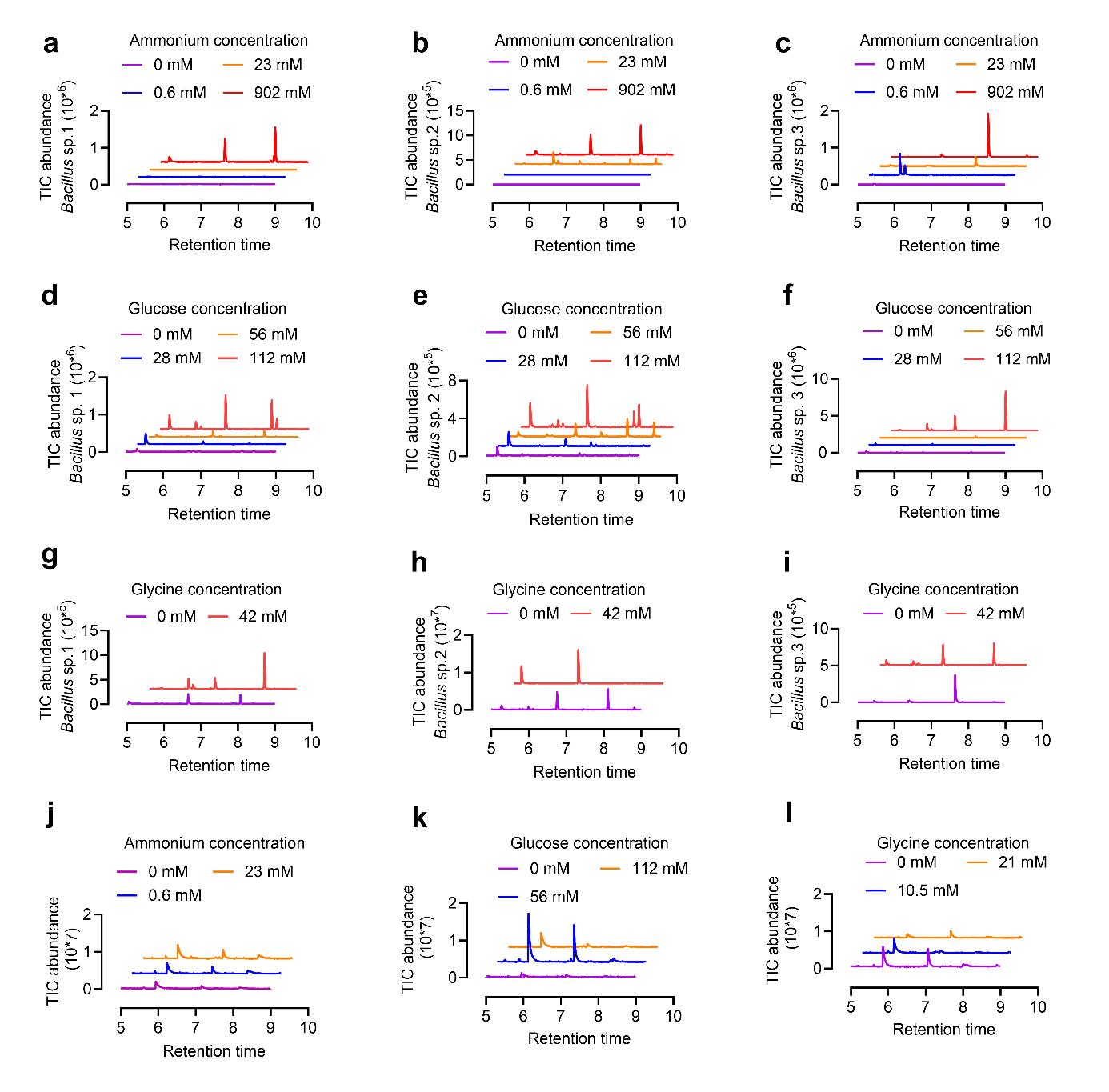


**Figure S3 GC-MS ion chromatograms of volatiles produced by *Bacillus* strains in culture medium with different ammonium (a-c), glucose (d-f), glycine (g-i) levels. GC-MS ion chromatograms of rectal volatiles after feeding different concentrations of ammonium (j), glucose (k), and glycine (l).**

**
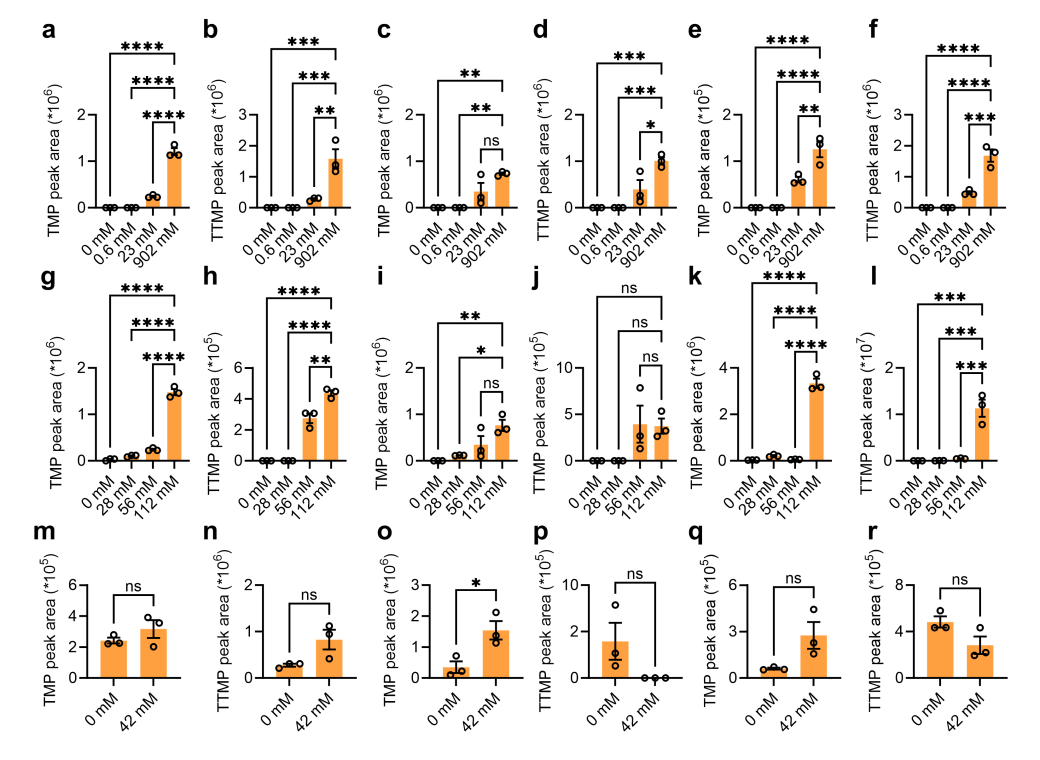
**

**Figure S4 Sex pheromone produced by *Bacillus* strains in culture medium with different glucose, glycine and ammonium levels.** (a) TMP produced by *Bacillus* sp.1 in culture medium with different ammonium levels (n = 3, *F*_(3,8)_ = 220, *P* < 0.0001, one-way ANOVA). (b) TTMP produced by *Bacillus* sp.1 in culture medium with different ammonium levels (n = 3, *F*_(3,8)_ = 23.78, *P* = 0.0002, one-way ANOVA). (c) TMP produced by *Bacillus* sp.2 in culture medium with different ammonium levels (n = 3, *F*_(3,8)_ = 13.45, *P* = 0.0017, one-way ANOVA). (d) TTMP produced by *Bacillus* sp.2 in culture medium with different ammonium levels (n = 3, *F*_(3,8)_ = 19.59, *P* = 0.0005, one-way ANOVA). (e) TMP produced by *Bacillus* sp.3 in culture medium with different ammonium levels (n = 3, *F*_(3,8)_ = 43.29, *P* < 0.0001, one-way ANOVA). (f) TTMP produced by *Bacillus* sp.3 in culture medium with different ammonium levels (n = 3, *F*_(3,8)_ = 58.65, *P* < 0.0001, one-way ANOVA). (g) TMP produced by *Bacillus* sp.1 in culture medium with different glucose levels (n = 3, *F*_(3,8)_ = 377, *P* < 0.0001, one-way ANOVA). (h) TTMP produced by *Bacillus* sp.1 in culture medium with different glucose levels (n = 3, *F*_(3,8)_ = 140.3, *P* < 0.0001, one-way ANOVA). (i) TMP produced by *Bacillus* sp.2 in culture medium with different glucose levels (n = 3, *F*_(3,8)_ = 9.078, *P* = 0.0059, one-way ANOVA). (j) TTMP produced by *Bacillus* sp.2 in culture medium with different glucose levels (n = 3, *F*_(3,8)_ = 4.24, *P* = 0.0454, one-way ANOVA). (k) TMP produced by *Bacillus* sp.3 in culture medium with different glucose levels (n = 3, *F*_(3,8)_ = 253.4, *P* < 0.0001, one-way ANOVA). (l) TTMP produced by *Bacillus* sp.3 in culture medium with different glucose levels (n = 3, *F*_(3,8)_ = 36.34, *P* < 0.0001, one-way ANOVA). (m) TMP produced by *Bacillus* sp.1 in culture medium with different glycine levels (n = 3, *P* = 0.2891, Independent sample *t* test). (n) TTMP produced by *Bacillus* sp.1 in culture medium with different glycine levels (n = 3, *P* = 0.0619, Independent sample *t* test). (o) TMP produced by *Bacillus* sp.2 in culture medium with different glycine levels (n = 3, *P* = 0.0276, Independent sample *t* test). (p) TTMP produced by *Bacillus* sp.2 in culture medium with different glycine levels (n = 3, *P* = 0.119, Independent sample *t* test). (q) TMP produced by *Bacillus* sp.3 in culture medium with different glycine levels (n = 3, *P* = 0.0677, Independent sample *t* test). (r) TTMP produced by *Bacillus* sp.3 in culture medium with different glycine levels (n = 3, *P* = 0.0899, Independent sample *t* test).


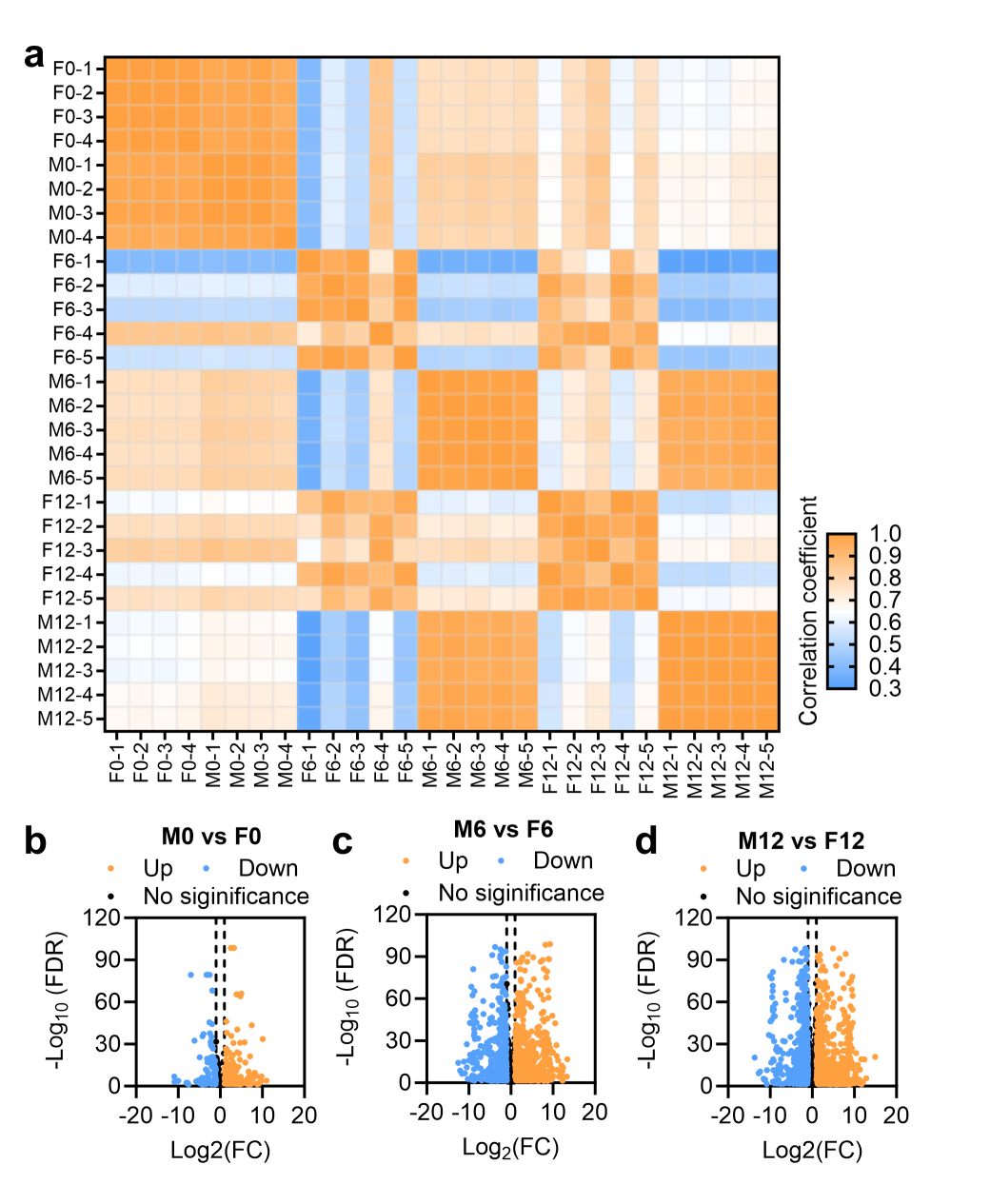


**Figure S5 Rectal transcriptome difference between male and female.** (a) Pearson correlation analysis showing differences between rectal gene expression patterns of male and female. (b) Expression fold changes of genes in the rectum of 0-day-old male compared with that of 0-day-old female. (c) Expression fold changes of genes in the rectum of 6-day-old male compared with that of 6-day-old female. (d) Expression fold changes of genes in the rectum of 12-day-old male compared with that of 12-day-old female.


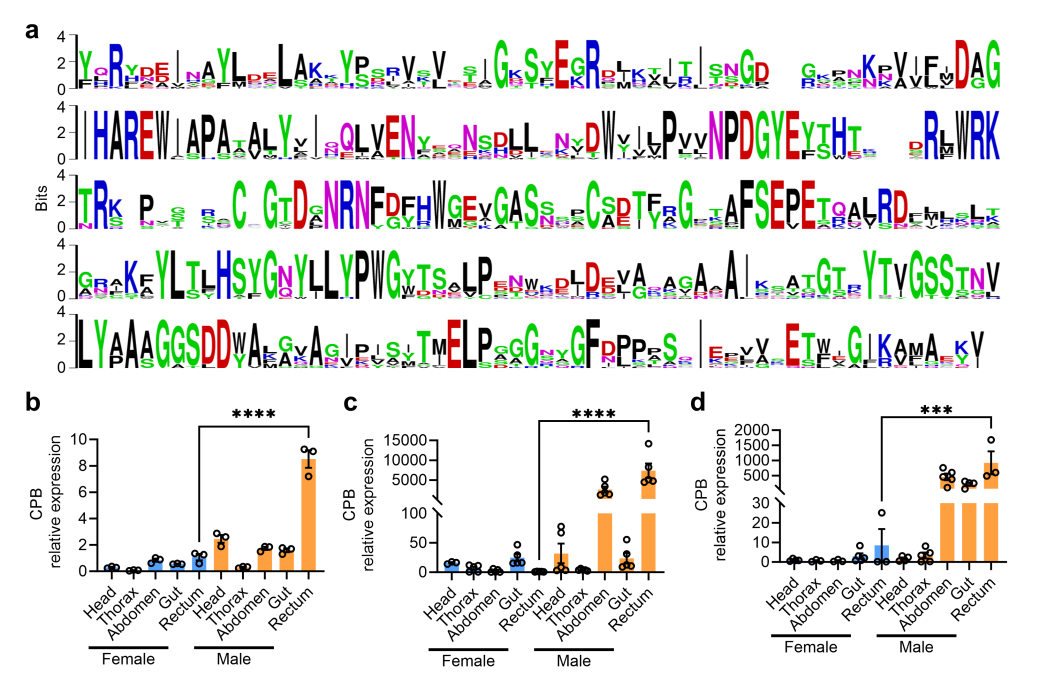


**Figure S6 Conservative and expression analysis of CPB.** (a) Conservation of the CPB protein in insects. The logo was generated using the mature region of 20 proteins from the species in Figure 4b. (b) Tissue expression of CPB in 0-day-old male and female (n = 3, *F*_(9,20)_ = 97.64, *P* < 0.0001, one-way ANOVA). (c) Tissue expression of CPB in 6-day-old male and female (n = 5, *F*_(9,38)_ = 13.62, *P* < 0.0001, one-way ANOVA). (d) Tissue expression of CPB in 12-day-old male and female (n = 3-5, *F*_(9,29)_ = 8.063, *P* < 0.0001, one-way ANOVA).


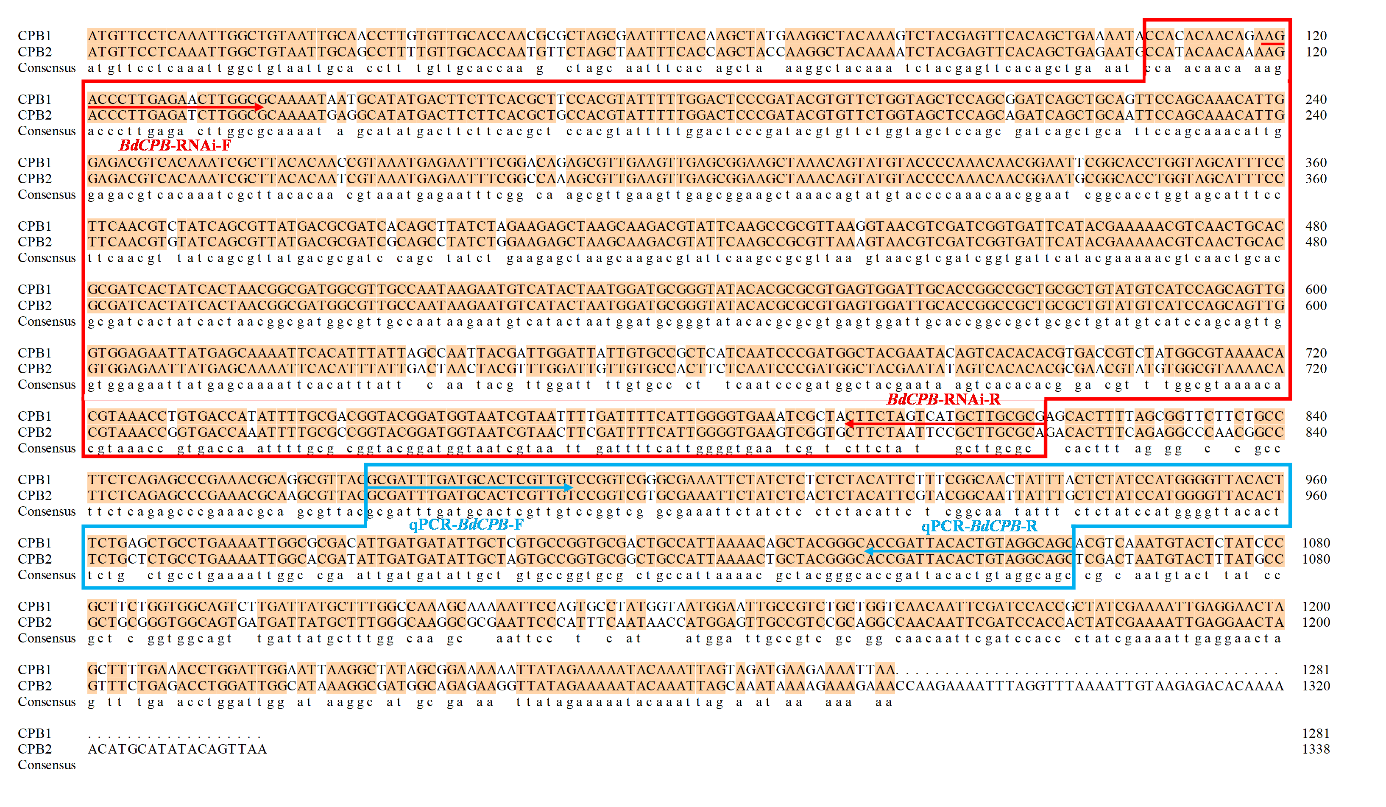


**Figure S7 mRNA sequences alignment for CPBs identified in male.** The sequence in the red box is the area where the dsRNA is designed and the sequence in the blue box is the area where qPCR is performed.


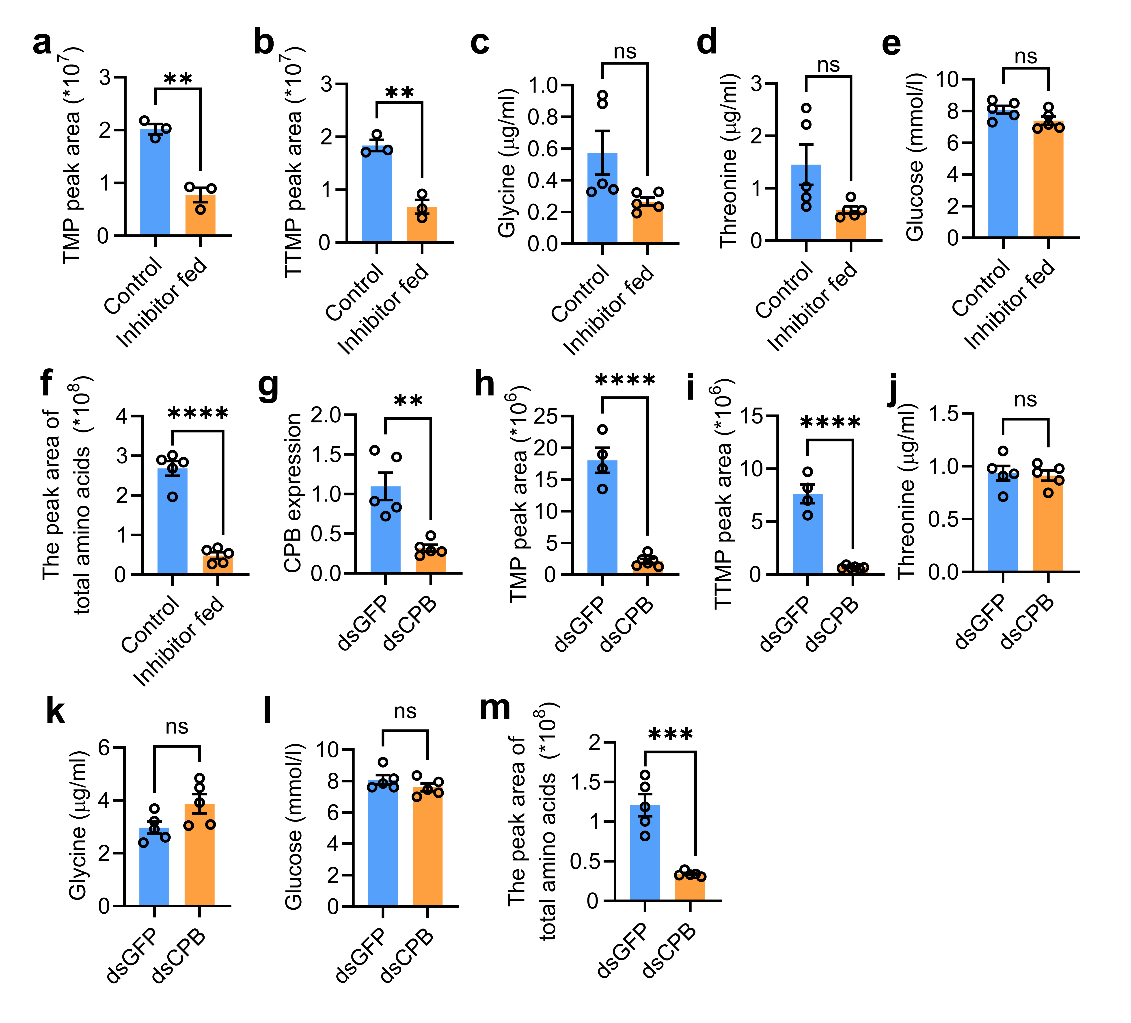


**Figure S8** **CPB regulates sex pheromone synthesis in rectum.** (a) Influence of CPB inhibitor feeding on TMP level (n = 3, *P* = 0.0018, Independent sample student’s *t* test). (b) Influence of CPB inhibitor feeding on TTMP level (n = 3, *P* = 0.0024, Independent sample student’s *t* test). (c) Influence of CPB inhibitor feeding on glycine level (n = 5, *P* = 0.0595, Independent sample student’s *t* test). (d) Influence of CPB inhibitor feeding on threonine level (n = 5, *P* = 0.058, Independent sample student’s *t* test). (e) Influence of CPB inhibitor feeding on glucose level (n = 5, *P* = 0.0925, Independent sample student’s *t* test). (f) Influence of CPB inhibitor feeding on total amino acids level (n = 5, *P* < 0.0001, Independent sample student’s *t* test). (g) CPB RNAi efficiency (n = 5, *P* = 0.0023, Independent sample student’s *t* test) (h) Influence of CPB knocking down on TMP level (n = 4 and 6, *P* < 0.0001, Independent sample student’s *t* test). (i) Influence of CPB knocking down on TTMP level (n = 4 and 6, *P* < 0.0001, Independent sample student’s *t* test). (j) Influence of CPB knocking down on threonine level (n = 5, *P* = 0.8023, Independent sample student’s *t* test). (k) Influence of CPB knocking down on glycine level (n = 5, *P* = 0.0656, Independent sample student’s *t* test). (l) Influence of CPB knocking down on glucose level (n = 5, *P* = 0.2476, Independent sample student’s *t* test). (m) Influence of CPB knocking down on total amino acids level (n = 5, *P* = 0.0003, Independent sample student’s *t* test).

**
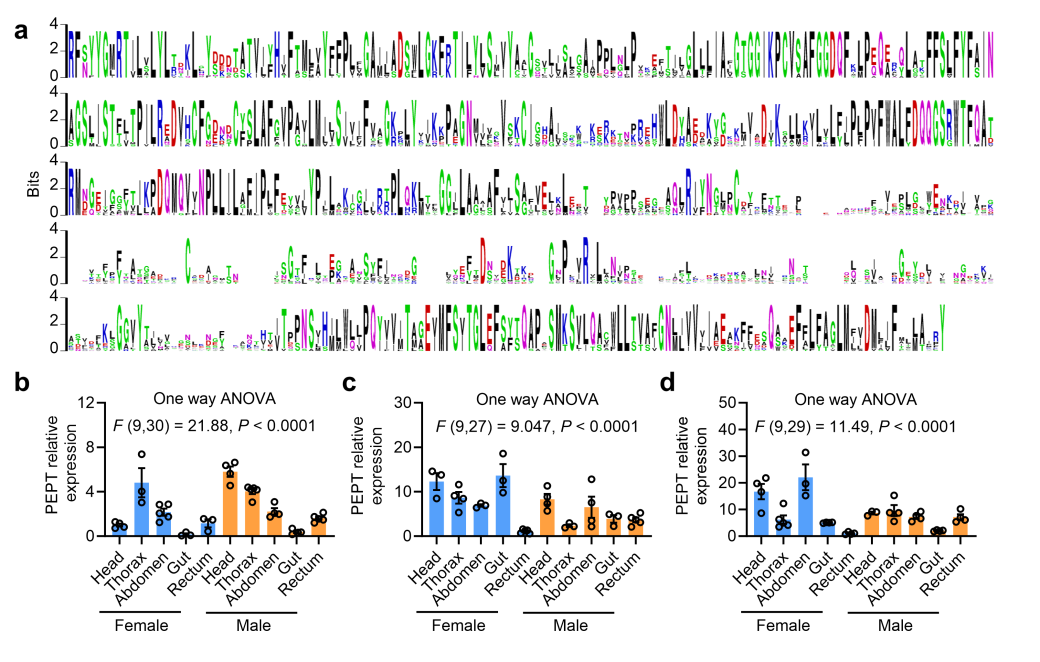
**

**Figure S9 Conservative and expression analysis of PEPT.** (a) Conservation of the PEPT protein in insects. The logo was generated using the mature region of 17 proteins from the species in Figure 5a. (b) Tissue expression of PEPT in 0-day-old male and female (n = 3-5, *F*_(9,30)_ = 21.88, *P* < 0.0001, one-way ANOVA). (c) Tissue expression of PEPT in 6-day-old male and female (n = 3-5, *F*_(9,27)_ = 9.047, *P* < 0.0001, one-way ANOVA). (d) Tissue expression of PEPT in 12-day-old male and female (n = 3-5, *F*_(9,29)_ = 11.49, *P* < 0.0001, one-way ANOVA).


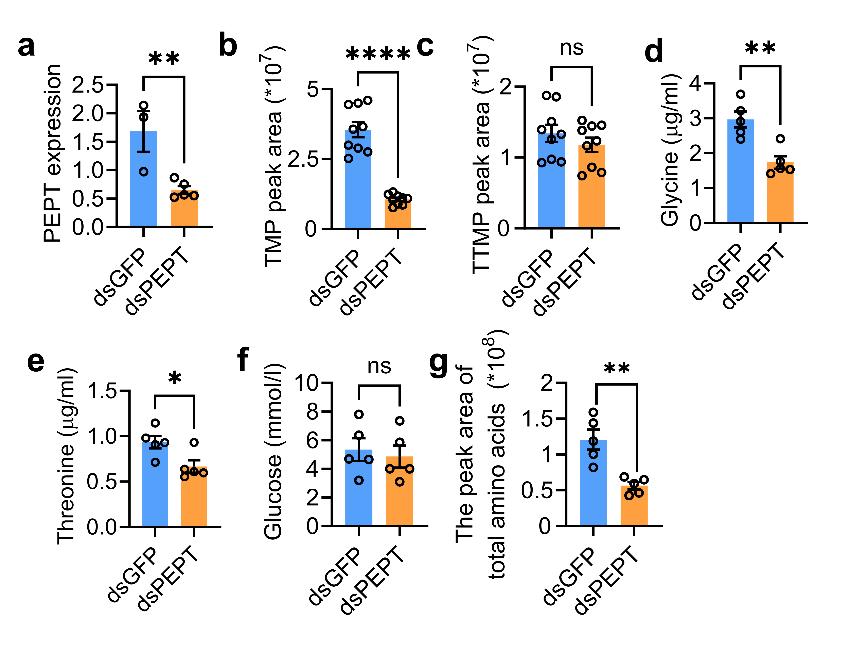


**Figure S10 Effect of PEPT knocking down on sex pheromone, glycine, threonine and glucose levels in rectum.** (a) PEPT knocking down efficiency (n = 3 and 5, *P* = 0.0099, Independent sample *t* test). (b) Influence of PEPT knocking down on rectum TMP level (n = 9, *P* < 0.0001, Independent sample student’s *t* test). (c) Influence of PEPT knocking down on rectum TTMP level (n = 9, *P* = 0.3218, Independent sample student’s *t* test). (d) Influence of PEPT knocking down on rectum glycine level (n = 5, *P* = 0.0028, Independent sample student’s *t* test). (e) Influence of PEPT knocking down on rectum threonine level (n = 5, *P* = 0.0242, Independent sample student’s *t* test). (f) Influence of PEPT knocking down on rectum glucose level (n = 5, *P* = 0.6747, Independent sample student’s *t* test). (g) Influence of PEPT knocking down on rectum total amino acids level (n = 5, *P* = 0.0025, Independent sample student’s *t* test).


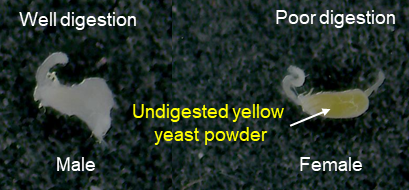


**Figure S11 Comparison of residual food digestibility between the rectum of mature male and female.**

**Table S1 Amino acid levels in rectal of mature female and male**

| Amino acids | Female (ug/ml) | Male (ug/ml) | *P* value | *t* value |
| --- | --- | --- | --- | --- |
| Taurine | 2.58425580±0.089490 | 2.704048±0.086851 | 0.365 | -0.961 |
| Phenethylamine | 0.954113±0.034970 | 0.888155±0.053665 | 0.333 | 0.272 |
| Aspartic acid | 1.32096±0.073877 | 3.459041±0.177236 | 0.000 | -11.135 |
| Serine | 2.071522±0.043076 | 2.172769±0.127579 | 0.474 | -0.752 |
| Asparagine | 1.166097±0.222067 | 1.320591±0.265319 | 0.667 | -0.447 |
| Glutamate | 9.74636±0.291520 | 13.738010±0.902975 | 0.003 | -4.207 |
| Glutamine | 17.32225±1.009716 | 37.015077±2.535425 | 0.000 | -7.216 |
| Alanine | 11.655938±0.398622 | 29.889417±1.938463 | 0.001 | -9.213 |
| Valine | 1.294063±0.072078 | 0.744754±0.036989 | 0.000 | 6.780 |
| Methionine | 0.294259±0.023488 | 0.246144±0.011233 | 0.102 | 1.848 |
| Cystathionine | 0.028435±0.002310 | 0.089161±0.012376 | 0.007 | -4.283 |
| Isoleucine | 1.004289±0.050489 | 0.603195±0.037545 | 0.000 | 6.375 |
| Leucine | 1.424072±0.053407 | 1.483119±0.062493 | 0.493 | -0.718 |
| Tyrosine | 1.526491±0.062321 | 1.214629±0.071731 | 0.002 | 4.531 |
| Phenylalanine | 0.557244±0.024954 | 0.438065±0.023516 | 0.008 | 3476.000 |
| β-Alanine | 2.137566±0.058971 | 0.849486±0.054964 | 0.000 | 15.978 |
| β-aminoisobutyric acid | 0.741469±0.036036 | 0.308110±0.014656 | 0.000 | 11.140 |
| γ－aminobutyric acid | 0.432937±0.046207 | 0.250507±0.025108 | 0.008 | 3.469 |
| Tryptophan | 0±0 | 9.5209196±1.221355 | < 0.0001 | 17.43 |
| Ethanolamine | 1.311717±0.058415 | 0.597475±0.058084 | 0.000 | 8.67 |
| Lysine | 1.876908±0.084964 | 0.696964±0.049098 | 0.000 | 12.024 |
| Histidine | 3.282885±0.064731 | 2.847033±0.144922 | 0.025 | 2.746 |
| Arginine | 9.581553±0.267386 | 6.941649±0.303178 | 0.000 | 6.531 |
| Proline | 11.412324±0.311091 | 11.771266±0.743545 | 0.668 | -0.445 |
| Glycine | 4.064954±0.106347 | 2.661533±0.117548 | < 0.0001 | 8.853 |
| Threonine | 0.990103±0.036418 | 0.972956±0.043656 | 0.7706 | 0.3016 |

**Table S2 Primers used in this study**

| Primer name | Primer sequence (5’-3’) |
| --- | --- |
| qPCR-Bacillus-F | TGAAACTYAAAGGAATTGACG |
| qPCR-Bacillus-R | ACCATGCACCACCTGTC |
| *BdCPB-*RNAi-F | AGACCCTTGAGAACTTGGCG |
| *BdCPB-*RNAi-R | TCGCGCAAGCATGACTAGAA |
| T7-*BdCPB-*RNAi-F | ggatcctaatacgactcactataggAGACCCTTGAGAACTTGGCG |
| T7-*BdCPB-*RNAi-R | ggatcctaatacgactcactataggTCGCGCAAGCATGACTAGAA |
| qPCR-*BdCPB*-F | GCGATTTGATGCACTCGTTGT |
| qPCR-*BdCPB*-R | GCTGCCTACAGTGTAATCGGT |
| *BdPEPT-*RNAi-F | GTTTGCTAGCCGCTATTGCC |
| *BdPEPT-*RNAi-R | TCAGAGTGAACGCACTGTCC |
| T7- *BdPEPT-*RNAi-F | ggatcctaatacgactcactatagg GTTTGCTAGCCGCTATTGCC |
| T7- *BdPEPT-*RNAi-R | ggatcctaatacgactcactatagg TCAGAGTGAACGCACTGTCC |
| qPCR-*BdPEPT*-F | TTGGCCACATTCTTCTCGCT |
| qPCR-*BdPEPT*-R | GAGCAACATAAGCATGGCCG |
| *GFP*-RNAi-F | ACTACCTGTTCCATGGCCAAC |
| *GFP*-RNAi-R | GAAAGGGCAGATTGTGTGGAC |
| T7-*GFP*-RNAi-F | ggatcctaatacgactcactatagg ACTACCTGTTCCATGGCCAAC |
| T7-*GFP*-RNAi-R | ggatcctaatacgactcactatagg GAAAGGGCAGATTGTGTGGAC |
| qPCR- *BdTUB* - F | CGCATTCATGGTTGATAACG |
| qPCR- *BdTUB* - R | GGGCACCAAGTTAGTCTGGA |
| qPCR- *BdRPL* - F | CGATTTCTCCGCAGTATTCAC |
| qPCR- *BdRPL* - R | GCCAGTACCTCATGCCTAACA |

**Table S3 Amino acid levels in rectal of mature male fed CPB inhibitor**

| amino acids | Control | PCI (inhibitor) | *P* value | *t* value |
| --- | --- | --- | --- | --- |
| Taurine | 1.948939±0.690651 | 0.655768±0.018432 | 0.134 | 1.87 |
| Phenethylamine | 1.548108±0.317333 | 0.479436±0.024013 | 0.028 | 3.358 |
| Aspartic acid | 3.764539±0.212079 | 1.205006±0.194723 | 0 | 8.89 |
| Serine | 1.503768±0.468708 | 0.944541±0.071451 | 0.301 | 1.179 |
| Asparagine | 1.675579±0.221243 | 0.665856±0.076545 | 0.003 | 4.313 |
| Glutamate | 9.388094±2.436838 | 5.084024±0.171064 | 0.152 | 1.762 |
| Glutamine | 28.707234±5.743292 | 25.741200±2.122321 | 0.684 | 0.484 |
| Alanine | 16.262293±2.877609 | 13.944234±1.601870 | 0.507 | 0.704 |
| Valine | 0.516384±0.080145 | 0.460214±0.033755 | 0.536 | 0.646 |
| Methionine | 0.157409±0.012555 | 0.096879±0.009439 | 0.008 | 3.674 |
| Isoleucine | 0.453938±0.053963 | 0.279746±0.020075 | 0.016 | 3.025 |
| Leucine | 1.083463±0.104309 | 0.472065±0.043494 | 0.002 | 5.41 |
| Tyrosine | 0.916757±0.257254 | 0.773540±0.051595 | 0.612 | 0.546 |
| Phenylalanine | 0.291602±0.050025 | 0.166927±0.026944 | 0.06 | 2.194 |
| β-Alanine | 0.511331±0.142080 | 0.266204±0.030451 | 0.161 | 1.687 |
| β-aminoisobutyric acid | 0.529255±0.053016 | 0.135532±0.012972 | 0.001 | 7.214 |
| γ－aminobutyric acid | 0.736356±0.266301 | 0.114001±0.013706 | 0.08 | 2.334 |
| Tryptophan | 6.625195±0.784515 | 0.242617±0.095587 | 0.001 | 8.076 |
| Ethanolamine | 0.098868±0.012057 | 0.052403±0.010133 | 0.018 | 2.950 |
| Lysine | 0.694376±0.173788 | 0.602482±0.025301 | 0.627 | 0.523 |
| Histidine | 1.916925±0.627067 | 1.438682±0.046721 | 0.489 | 0.761 |
| Arginine | 4.162146±1.671018 | 1.724802±0.168792 | 0.219 | 1.451 |
| Proline | 5.511317±1.794380 | 2.411303±0.215304 | 0.159 | 1.715 |
| Glycine | 0.573417±0.137920 | 0.265328±0.026067 | 0.0595 | 2.195 |
| Threonine | 1.452197±0.385099 | 0.587627±0.06760 | 0.0580 | 2.211 |

**Table S4 Amino acid levels in rectal after silencing CPB**

| Amino acids | ds*CPB* (ug/ml) | ds*GFP*(ug/ml) | *P* value | t value |
| --- | --- | --- | --- | --- |
| Taurine | 0.065366±0.042201 | 0.494573±0.065366 | 0.607 | -0.535 |
| Phenethylamine | 0.383130±0.120575 | 0.279547±0.024931 | 0.444 | -0.841 |
| Aspartic acid | 0.614404±0.090475 | 0.667608±0.073659 | 0.66 | 0.456 |
| Serine | 0.906200±0.040621 | 0.798899±0.039324 | 0.094 | -1.898 |
| Asparagine | 0.831882±0.069068 | 0.735148±0.052322 | 0.297 | -1.116 |
| Glutamate | 5.582910±1.344116 | 8.198359±0.540648 | 0.109 | 1.805 |
| Glutamine | 13.854058±0.647938 | 13.546943±0.992533 | 0.802 | -0.259 |
| Alanine | 11.585164±1.591449 | 13.185026±1.442092 | 0.478 | 0.745 |
| Valine | 0.428332±0.022396 | 0.440216±0.035431 | 0.784 | 0.284 |
| Methionine | 0.068477±0.006066 | 0.093570±0.014509 | 0.149 | 1.596 |
| Cystathionine | 0.047403±0.006849 | 0.078705±0.008242 | 0.019 | 2.921 |
| Isoleucine | 0.271972±0.018014 | 0.260829±0.022863 | 0.712 | -0.383 |
| Leucine | 0.537105±0.068589 | 0.779034±0.068183 | 0.037 | 2.502 |
| Tyrosine | 0.563156±0.031838 | 0.540392±0.049787 | 0.710 | -0.385 |
| Phenylalanine | 0.223524±0.041775 | 0.225218±0.021264 | 0.972 | 0.036 |
| β-Alanine | 0.410546±0.065284 | 0.345667±0.027150 | 0.386 | -0.918 |
| β-aminoisobutyric acid | 0.094069±0.017933 | 0.143445±0.017420 | 0.084 | 1.975 |
| γ－aminobutyric acid | 0.254752±0.101657 | 0.106297±0.008298 | 0.218 | -1.456 |
| Tryptophan | 0.451694±0.117000 | 5.199983±1.282290 | 0.006 | 3.688 |
| Ethanolamine | 0.400826±0.101505 | 0.200158±0.077692 | 0.155 | -1.570 |
| Lysine | 0.593792±0.062098 | 0.394472±0.048630 | 0.035 | 0.817 |
| Histidine | 1.070673±0.079274 | 0.860383±0.047077 | 0.052 | -2.281 |
| Arginine | 1.646337±0.167503 | 1.419578±0.137404 | 0.326 | -1.047 |
| Proline | 4.226052±0.306147 | 4.819507±0.435726 | 0.297 | 1.114 |
| Glycine | 3.877660±0.360832 | 2.967280±0.228265 | 0.656 | 2.1320 |
| Threonine | 0.912769±0.048452 | 0.93465600±0.069307 | 0.8023 | 0.2588 |

**Table S5 Amino acid levels in rectum after silencing PEPT**

| Amino acids | Ds*PEPT* (ug/ml) | ds*GFP*(ug/ml) | P value | t value |
| --- | --- | --- | --- | --- |
| Taurine | 0.789191±0.033699 | 0.494573±0.065366 | 0.004 | -4.006 |
| Phenethylamine | 0.299160±0.033238 | 0.279547±0.024931 | 0.649 | -0.472 |
| Aspartic acid | 1.257600±0.201554 | 0.667608±0.073659 | 0.025 | -2.749 |
| Serine | 0.960281±0.076008 | 0.798899±0.039324 | 0.096 | -1.886 |
| Asparagine | 0.655651±0.075151 | 0.735148±0.052322 | 0.411 | 0.868 |
| Glutamate | 6.094924±0.298530 | 8.198359±0.540648 | 0.009 | 3.406 |
| Glutamine | 20.766419±1.021354 | 13.546943±0.992533 | 0.001 | -5.069 |
| Alanine | 11.533712±0.415390 | 13.185026±1.442092 | 0.325 | 1.100 |
| Valine | 0.229840±0.021086 | 0.440216±0.035431 | 0.001 | 5.102 |
| Methionine | 0.051721±0.004934 | 0.093570±0.014509 | 0.026 | 2.731 |
| Cystathionine | 0.019072±0.005137 | 0.078705±0.008242 | 0.000 | 6.140 |
| Isoleucine | 0.172937±0.033488 | 0.260829±0.022863 | 0.062 | 2.168 |
| Leucine | 0.390016±0.045190 | 0.779034±0.068183 | 0.001 | 0.379 |
| Tyrosine | 0.391601±0.028093 | 0.540392±0.049787 | 0.031 | 2.603 |
| Phenylalanine | 0.160122±0.027790 | 0.225218±0.021264 | 0.100 | 1.860 |
| β-Alanine | 0.506855±0.037018 | 0.345667±0.027150 | 0.008 | -3.511 |
| β-aminoisobutyric acid | 0.089919±0.003722 | 0.143445±0.017420 | 0.017 | 3.005 |
| γ－aminobutyric acid | 0.306930±0.164969 | 0.106297±0.008298 | 0.291 | -1.215 |
| Tryptophan | 1.997443±0.341074 | 5.199983±1.282290 | 0.042 | 2.414 |
| Ethanolamine | 0.305726±0.091216 | 0.200158±0.077692 | 0.404 | -0.881 |
| Lysine | 0.353623±0.043610 | 0.394472±0.048630 | 0.549 | 0.625 |
| Histidine | 1.189122±0.057693 | 0.860383±0.047077 | 0.002 | -4.415 |
| Arginine | 2.999757±0.128282 | 1.419578±0.137404 | 0.000 | -8.406 |
| Proline | 5.247311±0.294542 | 4.819507±0.435726 | 0.440 | -0.813 |
| Glycine | 1.736740±0.178304 | 2.967280±0.228265 | 0.0028 | 4.248 |
| Threonine | 0.665636±0.067889 | 0.934656±0.069307 | 0.0242 | 2.773 |

**Supplementary Datasets**

**Dataset S1 Rectal bacteria expression profile.**

**Dataset S2 Gene expression profile in rectum of male and female at different development stages.**

**Dataset S3 DE genes between 0-day-old male and 0-day-old female.**

**Dataset S4 DE genes between 6-day-old male and 6-day-old female.**

**Dataset S5 DE genes between 12-day-old male and 12-day-old female.**

**Dataset S6 Pathway enriched with DE genes between 6d female and 6d male.**

**Dataset S7 DE genes in protein digestion and absorption.**

**Dataset S8 Pathway enriched with DE genes between 12d female and 12d male.**

**Reference**

1. Khoo, C. C. & Tan, K. H. Rectal gland of *Bactrocera papayae*: ultrastructure, anatomy, and sequestration of autofluorescent compounds upon methyl eugenol consumption by the male fruit fly. *Microsc Res Tech* **67**, 219-226 (2005). https://doi.org/10.1002/jemt.20199
